# Distinct Volume Alterations of Thalamic Nuclei Across the Schizophrenia Spectrum

**DOI:** 10.1093/schbul/sbae037

**Published:** 2024-04-05

**Authors:** Melissa Thalhammer, Julia Schulz, Felicitas Scheulen, Mohamed El Mehdi Oubaggi, Matthias Kirschner, Stefan Kaiser, André Schmidt, Stefan Borgwardt, Mihai Avram, Felix Brandl, Christian Sorg

**Affiliations:** Department of Diagnostic and Interventional Neuroradiology, School of Medicine, Technical University of Munich, Munich, Germany; TUM-NIC Neuroimaging Center, School of Medicine, Technical University of Munich, Munich, Germany; Department of Diagnostic and Interventional Neuroradiology, School of Medicine, Technical University of Munich, Munich, Germany; TUM-NIC Neuroimaging Center, School of Medicine, Technical University of Munich, Munich, Germany; Department of Diagnostic and Interventional Neuroradiology, School of Medicine, Technical University of Munich, Munich, Germany; TUM-NIC Neuroimaging Center, School of Medicine, Technical University of Munich, Munich, Germany; Department of Diagnostic and Interventional Neuroradiology, School of Medicine, Technical University of Munich, Munich, Germany; TUM-NIC Neuroimaging Center, School of Medicine, Technical University of Munich, Munich, Germany; Department of Psychiatry, University Hospital of Geneva, Geneva, Switzerland; Department of Psychiatry, Psychotherapy and Psychosomatics, Psychiatric Hospital, University of Zurich, Zurich, Switzerland; Department of Psychiatry, University Hospital of Geneva, Geneva, Switzerland; Department of Psychiatry (UPK), University of Basel, Basel, Switzerland; Translational Psychiatry, Department of Psychiatry and Psychotherapy, University of Lübeck, Lübeck, Germany; Translational Psychiatry, Department of Psychiatry and Psychotherapy, University of Lübeck, Lübeck, Germany; Department of Diagnostic and Interventional Neuroradiology, School of Medicine, Technical University of Munich, Munich, Germany; TUM-NIC Neuroimaging Center, School of Medicine, Technical University of Munich, Munich, Germany; Department of Psychiatry and Psychotherapy, School of Medicine, Technical University of Munich, Munich, Germany; Department of Diagnostic and Interventional Neuroradiology, School of Medicine, Technical University of Munich, Munich, Germany; TUM-NIC Neuroimaging Center, School of Medicine, Technical University of Munich, Munich, Germany; Department of Psychiatry and Psychotherapy, School of Medicine, Technical University of Munich, Munich, Germany

**Keywords:** schizophrenia, thalamic nuclei, magnetic resonance imaging, cognitive symptoms

## Abstract

**Background and Hypothesis:**

Abnormal thalamic nuclei volumes and their link to cognitive impairments have been observed in schizophrenia. However, whether and how this finding extends to the schizophrenia spectrum is unknown. We hypothesized a distinct pattern of aberrant thalamic nuclei volume across the spectrum and examined its potential associations with cognitive symptoms.

**Study Design:**

We performed a FreeSurfer-based volumetry of T_1_-weighted brain MRIs from 137 healthy controls, 66 at-risk mental state (ARMS) subjects, 89 first-episode psychosis (FEP) individuals, and 126 patients with schizophrenia to estimate thalamic nuclei volumes of six nuclei groups (anterior, lateral, ventral, intralaminar, medial, and pulvinar). We used linear regression models, controlling for sex, age, and estimated total intracranial volume, both to compare thalamic nuclei volumes across groups and to investigate their associations with positive, negative, and cognitive symptoms.

**Study Results:**

We observed significant volume alterations in medial and lateral thalamic nuclei. Medial nuclei displayed consistently reduced volumes across the spectrum compared to controls, while lower lateral nuclei volumes were only observed in schizophrenia. Whereas positive and negative symptoms were not associated with reduced nuclei volumes across all groups, higher cognitive scores were linked to lower volumes of medial nuclei in ARMS. In FEP, cognition was not linked to nuclei volumes. In schizophrenia, lower cognitive performance was associated with lower medial volumes.

**Conclusions:**

Results demonstrate distinct thalamic nuclei volume reductions across the schizophrenia spectrum, with lower medial nuclei volumes linked to cognitive deficits in ARMS and schizophrenia. Data suggest a distinctive trajectory of thalamic nuclei abnormalities along the course of schizophrenia.

## Introduction

Schizophrenia is a severe mental disorder that is heritable, polygenic, and heterogeneous in symptoms as well as in their course.^1,2^ Symptoms cluster into positive (eg, psychotic), negative (eg, anhedonia or social withdrawal), and cognitive symptoms (eg, attention or executive function deficits). While negative and cognitive symptoms show a rather stable course from their usual onset in early adolescence, psychotic symptoms typically fluctuate between psychotic episodes and psychotic remission. Thus, “pre-stages” exist in which psychotic risks or episodes occur without the presence of a manifest disorder. An “at-risk mental state” (ARMS) for psychosis is defined as having either attenuated psychotic symptoms of varying duration or a strong genetic vulnerability to psychosis, indicating a likely conversion to psychosis.^3–5^ Individuals at risk for psychosis were found to have a 36% transition rate at 3 years,^6^ which is tremendously higher than a 1% lifetime risk in the general population.^2^ When psychotic symptoms appear for the first time, the occurrence is called “first episode psychosis” (FEP).^7^ ARMS, FEP, and established schizophrenia (SCZ) together are referred to as the “schizophrenia spectrum.”^3,8,9^

Among various structural brain changes compared to the healthy population, abnormalities in cortico-thalamic circuits have been consistently observed in both schizophrenia^10–14^ and in the pre-stages ARMS and FEP as well as in psychosis-spectrum youths.^15–17,18^ Thereby, thalamic nuclei act as central hubs within these circuits,^19–21^ suggesting that the thalamus is crucially involved in the pathophysiology of schizophrenia.^22–26^ Indeed, volumetric studies focusing on the whole thalamus have consistently demonstrated smaller volumes in patients.^27–29^ Additionally, recent studies began to acknowledge the heterogeneous structure and functions of the distinct thalamic nuclei.^30–32^ This has been facilitated by the recent development of a pipeline for segmenting thalamic nuclei on T1-weighted MR images.^33^ Results from several postmortem as well as in vivo imaging studies distill that specific higher-order thalamic nuclei, namely the medial, pulvinar, and lateral nuclei, show lower volumes in established schizophrenia compared to healthy controls, which are frequently associated with cognitive impairments.^30,32,34–37^ Particularly, lower mediodorsal thalamic nuclei volumes^38^ as well as aberrant prefrontal-mediodorsal thalamic connectivity^10,39–41^ have previously been linked to cognitive impairments in schizophrenia. Moreover, cortico-thalamic hypoconnectivity has been reported in ARMS.^42^ However, it remains to be elucidated whether thalamic nuclei volume reductions occur prior to disease manifestation since previous studies have focused mainly on one stage of the disorder and have not covered the entire spectrum of schizophrenia. In addition, there is scarce evidence for nuclei-based volumetric studies in the pre-stages of the disorders.

Thus, our study of thalamic nuclei volumes across the schizophrenia spectrum pursued the following objectives: First, based on previous findings, particularly in established schizophrenia, we hypothesized that only specific nuclei, primarily the medial and pulvinar subregions, exhibit lower volumes than the control group. Due to conflicting and incomplete results derived from previous literature investigating pre-chronic phases, we did not have a clear hypothesis regarding these stages. To examine our hypothesis, the volumes of thalamic nuclei were determined in three stages of the schizophrenia spectrum as well as in age- and sex-matched healthy controls using a pipeline commonly used in and validated by the neuroimaging community.^33^ Second, we sought to study a potential link between volumetric aberrations and distinct symptom dimensions, namely positive, negative, and cognitive, in the patient groups. Given prior, albeit inconsistent, evidence regarding the possible involvement of medial and pulvinar nuclei groups in cognitive impairments,^10,38–41^ we expected a significant association between altered medial subregion volume and cognitive deficits in schizophrenia.

## Materials and Methods

### Participants

Structural T_1_-weighted brain images and clinical data of 522 subjects were collected from four sites, comprising control participants without psychopathology (HC) as well as three stages of the schizophrenia spectrum: ARMS, FEP, and established SCZ. These data have been used previously to investigate orbitofrontal-striatal abnormalities in relation to negative symptoms^43^ as well as to assess basal-forebrain cholinergic nuclei alterations in relation to cognitive symptoms^44^ across the schizophrenia spectrum. See table 1 and Supplementary Methods for an overview and more detailed demographics of each group per scanning site.

**Table 1.** Demographics Per Acquisition Site

	Munich (*n* = 43)	COBRE (*n* = 115)	Zurich (*n* = 90)	Basel (*n* = 170)
HC				
*n*	23	51	25	38
Age				
Mean (*SD*)	38.78 (11.84)	37.00 (11.67)	31.28 (8.38)	25.03 (3.93)
Range	25–62	18–65	18–54	19–35
Sex				
*n* (%) females	14 (60.87)	13 (25.49)	9 (36.00)	22 (57.89)
ARMS				
*n*				66
Age				
Mean (*SD*)				25.02 (5.69)
Range				18–42
Sex				
*n* (%) females				18 (27.27)
FEP				
*n*			23	66
Age				
Mean (*SD*)			24.87 (7.26)	27.30 (7.06)
Range			19–48	18–47
Sex				
*n* (%) females			5 (21.74)	18 (27.27)
SCZ				
*n*	20	64	42	
Age				
Mean (*SD*)	42.65 (12.39)	38.55 (13.75)	33.53 (7.93)	
Range	23–65	18–64	19–49	
Sex				
*n* (%) females	12 (60.00)	11 (17.19)	11 (26.19)	

*Note*: ARMS, at-risk mental state; FEP, first-episode psychosis; HC, healthy control; SCZ, schizophrenia; SD, standard deviation.

Data are listed for the final data sample after exclusion due to quality control. Number of participants data are available for is denoted as *n*.

#### Munich Dataset.

26 patients with schizophrenia and 24 age- and sex-matched healthy controls were recruited by the Department of Psychiatry of Klinikum rechts der Isar, Munich, and have been analyzed previously.^45–47^ Patients were in psychotic remission according to DSM-IV-TR criteria.^48,49^ Exclusion criteria for healthy controls were a history of axis I disorder, substance abuse, and first-degree relatives with a history of psychosis. Substance abuse was eliminated via urine screening or clinical interview. Patients’ antipsychotic medication was kept stable for at least 2 weeks prior to the scan. Psychotic and negative symptoms were evaluated with the Positive and Negative Syndrome Scale (PANSS).^50^

#### COBRE Dataset.

An open-source dataset provided by the Center for Biomedical Research Excellence (COBRE) database (http://fcon_1000.projects.nitrc.org/indi/retro/cobre.html) comprising 72 schizophrenic patients meeting DSM-IV criteria^49^ and 73 healthy controls were incorporated into the analysis. Antipsychotic medication was kept stable for a minimum of 4 weeks before the study. HC had no history of DSM-IV axis I disorders or psychosis in any first-degree relative. Urine screening was Applied to detect substance abuse. Psychotic and negative symptoms were measured using PANSS.^50^

#### Zurich Dataset.

Data of 48 patients with schizophrenia, 26 FEP subjects, as well as 28 healthy controls were acquired and previously analyzed.^43,51,52^ Clinical diagnosis was based on the structured Mini-International Neuropsychiatric Interview for DSM-IV (M.I.N.I).^53^ The inclusion criterion for SCZ was a clinical diagnosis of schizophrenia. Subjects with FEP were recruited during their first psychiatric admission in outpatient (*n* = 6) and inpatient (*n* = 20) units of the Psychiatric Hospital of the University of Zurich.^52^ FEP was defined as having a first clinical diagnosis of brief psychotic disorder, schizophreniform disorder, or first-episode schizophrenia using M.I.N.I.^53^ FEP subjects with a positive subscale item higher than 5 on PANSS were excluded because the initial aim of the experiment was to study the link between striatal connectivity aberrations with negative symptoms.^43,51,52^ For all patients, antipsychotic medication was kept stable for a minimum of 2 weeks before the study. Patients were excluded if another DSM-IV axis I disorder was diagnosed, benzodiazepines (> 1 mg/day Lorazepam-equivalent) were taken, or if extrapyramidal side effects were evident. Exclusion criteria for HC were psychiatric disorders, history of psychiatric disorders, and substance abuse.

#### Basel Dataset.

Data from 77 FEP individuals, 73 ARMS subjects, and 44 healthy controls were retrieved from previous studies.^54,55^ All individuals potentially at risk for an early stage of schizophrenia were intensively assessed based on variables previously shown to be predictors of psychosis such as social decline or genetic risk.^56,57^ Each individual identified to be at-risk was followed up every 1–3 months during the first 3 years, then yearly, for a thorough examination of potential predictors of schizophrenia. In particular, ARMS for psychosis was defined based on the Basel Screening Instrument for Psychosis^58^ and the Brief Psychiatric Rating Scale (BPRS)^4,59^ as showing “attenuated” psychotic symptoms, brief limited intermittent psychotic symptoms, or having a first- or second-degree relative with psychotic disorder and a marked decline in social functioning. More precisely, the decline in social functioning was defined as a “marked deterioration of psychosocial functioning with serious consequences for work, education, relationships (occurrence during the last 5 years and persisting up to now).”^57^ After a clinical follow-up of 33.3 months, 15 ARMS individuals had transitioned to psychosis. First-episode psychosis included subjects who fulfilled the criteria for acute psychotic disorder according to the International Statistical Classification of Diseases and Related Health Problems, 10th Revision,^60^ or DSM-IV criteria.^49^ In particular, a BPRS^4,59^ score of ≥4 on the hallucination item, or ≥5 on the unusual thought content, suspiciousness item, or conceptual disorganization item were set as the threshold.^61,62^ Symptoms needed to be present at least several times a week and the change in mental state lasting >1 week.^57^ 27 FEP patients were medication-free, while 45 were prescribed antipsychotics. Exclusion criteria for HC were psychiatric disorders or history of psychiatric disorders, head trauma, neurological illness, serious medical illness, substance abuse, or psychiatric disorders in family history. The Scale for the Assessment of Negative Symptoms (SANS) was used to assess the severity of negative symptoms.^48^

In summary, 73 ARMS participants, 104 FEP participants, 146 patients with schizophrenia, as well as 146 control subjects were examined. Following quality control of MRI cortical and thalamic nuclei segmentation, the final cohort consisted of 66 ARMS participants, 89 FEP individuals, 126 patients with schizophrenia, and 137 healthy controls (see below). For detailed information about data-based exclusion criteria, see Supplementary Methods. A summary of all available measures as well as patient characteristics is given in Supplementary table S1.

All studies were approved by the local ethics committees and all participants provided written informed consent.

### Symptom Ratings: Positive, Negative, and Cognitive Symptoms

For schizophrenia patients and some FEP subjects, psychotic, and negative symptoms were evaluated with the Positive and Negative Syndrome Scale (PANSS).^50^ For ARMS and some FEP individuals, the Brief Psychiatric Rating Scale (BPRS)^61^ and Scale for the Assessment of Negative Symptoms (SANS)^48^ were used alternatively to assess positive and negative, or negative symptomatology, respectively. Refer to Supplementary table S1 for an overview.

As the four acquisition sites used different neuropsychological batteries for cognitive testing, several test scores were assessed to investigate patients’ cognitive abilities (Supplementary table S1). The Verbal Fluency Test was available for all patient groups as well as HC. It captures processing speed and semantic memory by asking participants to name as many words as possible in 60 s. Semantic fluency tasks involve words that belong to a specific category (here: animals; “animal fluency”), while phenological fluency tasks ask participants to name as many words as possible that begin with a specific letter (here: letter S).^63,64^ In addition, the Trail Making Test Part A (TMT-A) and the Symbol Coding Task of the Brief Assessment of Cognition in Schizophrenia (SCT) were available for SCZ and HC subjects only. In the TMT-A, processing speed and visual attention are assessed by measuring the time to join numbers with a line in seconds.^65^ Similarly, the SCT determines processing speed and attention by asking subjects to decode symbols into numbers with a given decoding key.^66^ For an overview of relevant cognitive processes as well as linked thalamic outcomes associated with the cognitive tasks conducted, see Supplementary table S2.

### Assessment of Clinical Characteristics

The assessment of several clinical characteristics was conducted by psychiatrists at each site. Current antipsychotic medication in terms of chlorpromazine (CPZ) equivalents was determined in milligrams per day (mg/day),^67^ whereas the cumulative effect of anticholinergics was determined in terms of anticholinergic burden of medication (ACB) defined in arbitrary units (a.u., see https://www.acbcalc.com/).^68^

### Image Acquisition, Processing, Volume Outcomes, and Harmonization

T_1_-weighted structural brain MRI scans were acquired at each site. MRI data acquisition parameters are described in detail in the Supplementary Methods section for each site.

Next, the scans were processed using FreeSurfer’s (v 7.1.1; https://surfer.nmr.mgh.harvard.edu/) cortical reconstruction process *recon-all*. In brief, artifact correction, skull stripping, normalization into standard space as well as cortical segmentation and parcellation were performed. After assessing reconstruction quality visually, following FreeSurfer recommendations (https://surfer.nmr.mgh.harvard.edu/fswiki/QATools), 25 individuals were excluded due to processing inaccuracies (see a detailed description of exclusion criteria in Supplementary Methods).

Afterwards, a built-in thalamus segmentation pipeline^33^ was applied to segment the thalamus into 26 nuclei per hemisphere. Quality inspection following recent standards of the community^69,70^ led to the exclusion of a further 50 participants due to unsatisfactory segmentation results. Detailed exclusion criteria and quality assessment procedures are described in the Supplementary material. Volumes in mm^3^ were extracted for each subject and each nucleus directly from FreeSurfer.

**Fig. 1.**
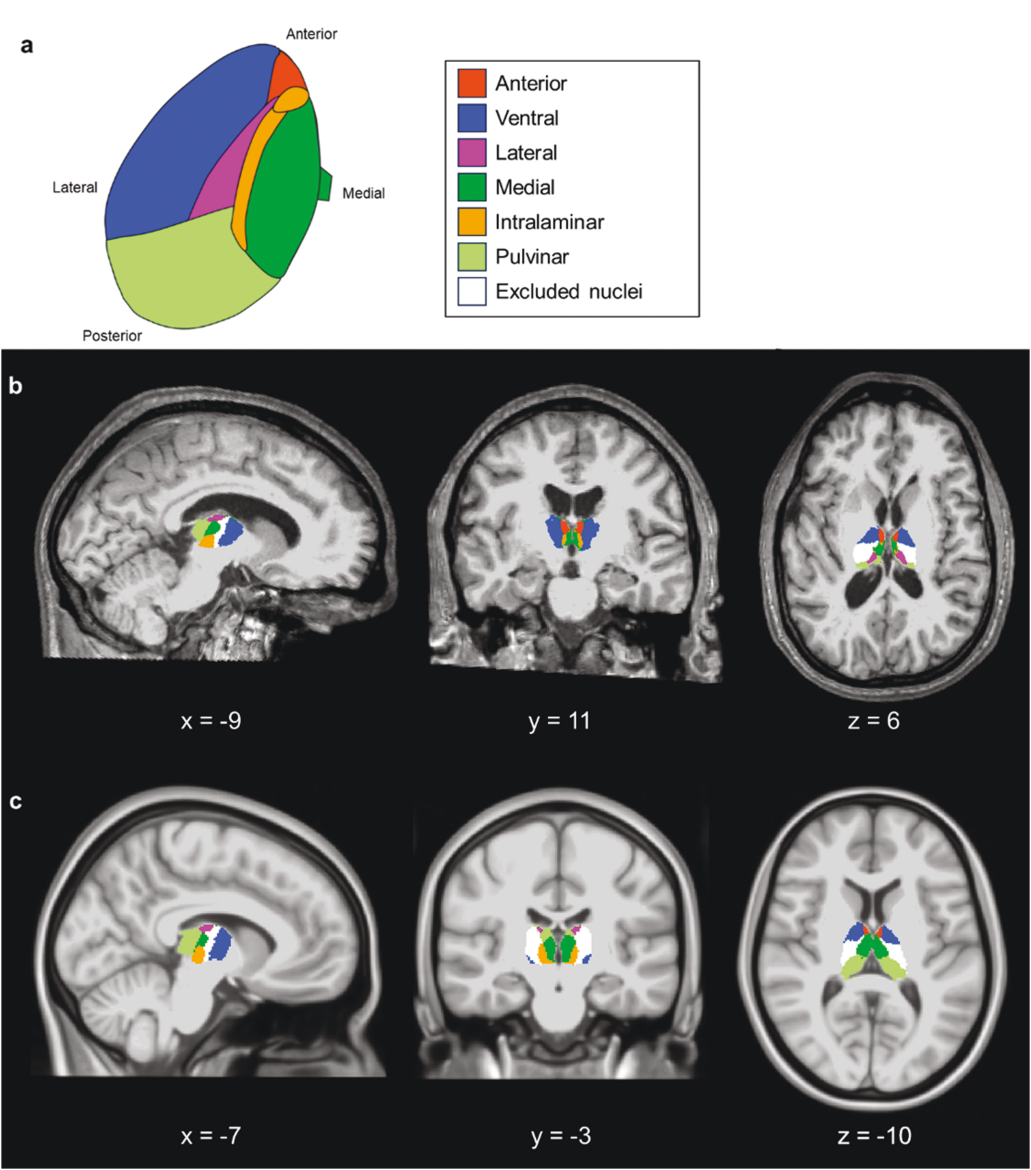
Thalamic nuclei grouping based on segmentation in FreeSurfer. The thalami are segmented into 26 nuclei for each hemisphere, as listed in table 2. (a) Based on literature recommendations,^33,69^ thalamic nuclei were then grouped into six nuclei clusters per hemisphere (see table 2): anterior (red), ventral (blue), lateral (pink), medial (dark green), intralaminar (orange), and pulvinar (light green). Excluded nuclei due to previous recommendations or overall poor segmentation quality are summarized and shown in white. (b) Depicts an actual segmentation performed on a 30-year-old healthy male of the COBRE dataset. In (c), thalamic subregions are overlaid on a bias-field corrected T1w image of the MNI152 template as obtained from FSL standards. MNI, Montreal Neurological Institute. Panel (a) was created with BioRender.com.

In accordance with prior literature,^33,69^ we grouped these nuclei into different subregions: anterior, lateral, ventral, intralaminar, medial, and pulvinar (see table 2 and figure 1).

**Table 2.** Thalamic Nuclei Grouping

Thalamic Regions	Included Thalamic Nuclei
Anterior	Anteroventral (AV)
Lateral	Laterodorsal (LD)
	Lateral posterior (LP)
Ventral	Ventral anterior (VA)
	Ventral anterior magnocellular (VAmc)
	Ventral lateral anterior (VLa)
	Ventromedial (VM)
Intralaminar	Central medial (CeM)
	Central lateral (CL)
	Paracentral (Pc)
	Centromedian (CM)
	Parafascicular (Pf)
Medial	Paratenial (Pt)
	Reuniens (medial ventral) (MV-re)
	Mediodorsal medial magnocellular (MDm)
	Mediodorsal lateral parvocellular (MDl)
Pulvinar	Pulvinar anterior (PuA)
	Pulvinar medial (PuM)
	Pulvinar lateral (PuL)
	Pulvinar inferior (PuI)

*Note*: According to suggestions in previous literature,^33,69^ thalamic subregions were defined incorporating the nuclei distinguished with the Thalamic Nuclei pipeline^33^ listed in the left column.

Since the naïve combination of neuroimaging data across different sites introduces variance mostly attributed to varying scanning protocols and hardware, volumetric outcomes were retrospectively harmonized for scanner effects using NeuroCombat (v0.12.2, in Python).^71^ Adopted from genomics,^72^ Combat models site-specific scaling factors by using an empirical Bayes method to adjust the data for batch effects while preserving a priori known sources of biological variance.^71^ The method has repeatedly shown great potential to remove scanner variation in diffusion tensor imaging,^73^ structural,^71,74^ and functional^75^ MRI data. Estimated total intracranial volume (eTIV) was corrected while preserving biological variation related to diagnosis, sex, and age, whereas eTIV variance was additionally retained when harmonizing subregional thalamic measures. Harmonization successfully removed variance attributed to the hardware (see Supplementary figure S1). However, since we are aware that there are limitations to NeuroCombat if sites acquired data from different groups,^76,77^ we compared subregion volumes within the Basel cohort before harmonization (Supplementary Results).

### Statistical analysis

#### Group Difference in Thalamic Subregion Volumes.

To estimate group differences, analyses of covariance (ANCOVA) were run on the harmonized data in IBM SPSS Statistics (IBM Corp. Released 2021. IBM SPSS Statistics for Windows, Version 28.0. Armonk, NY: IBM Corp). Mean volumes of the six thalamic subregions served as the within-subject variables, whereas group was the between-subject variable. Biological sex, age, and scanner-corrected eTIV were included as covariates of no interest. In particular, eTIV was included to control for individual head size differences among subjects. Fisher’s Least Significant Difference (LSD) test was applied for post hoc analyses separately for each test. Subsequently, Benjamini–Hochberg correction for multiple comparisons^78^ was applied to correct for the use of six different tests in total. Partial eta square (η*p*^2^) was used to estimate effect sizes.

#### Control Analyses for Group Difference in Thalamic Subregion Volumes.

Multiple linear regression models, as described in the following paragraph, were used to link reduced thalamic nuclei volumes with other confounding variables, namely medication, age, and illness duration. See Supplementary Methods for a detailed description.

#### Associations Between Thalamic Subregion Volumes and Cognitive Performance.

Differences in cognitive scores between groups were determined using an ANCOVA with the respective test as a dependent variable, group as a factor, and age and sex as covariates.

Testing the associations between cognitive performance and thalamic subregion volumes was conducted in SPSS using multiple linear regression analyses. To illustrate the effect of each covariate on the association between symptoms and subregional volume, we performed various regression analyses in a hierarchical framework, that is, we extended the model stepwise by one covariate. Thus, we came up with four different models to test the interaction between volume and positive, negative, and cognitive symptom scores (sym_pos_, sym_neg_, and sym_cog_, respectively):


$$\begin{array}{l} volume=\beta_{0}+\beta_{1}*sym_{pos}+\beta_{2}*sym_{neg}+\beta_{3}*sym_{cog} \end{array}$$

$$\begin{array}{lll} volume= & \;\beta_{0}+\beta_{1}¯sym_{pos}+\beta_{2}¯sym_{neg}\; & +\beta_{3}¯sym_{cog}+\beta_{4}¯sex \end{array}$$

$$\begin{array}{l} volume=\beta_{0}+\beta_{1}¯sym_{pos}+\beta_{2}¯sym_{neg}+\beta_{3}¯sym_{cog}+\beta_{4}¯sex+\beta_{5}¯eTIV \end{array}$$

$$\begin{array}{l} volume=\beta_{0}+\beta_{1}¯sym_{pos}+\beta_{2}¯sym_{neg}+\beta_{3}¯sym_{cog}+\beta_{4}¯sex+\beta_{5}¯eTIV+\beta_{6}¯age \end{array}$$

Subjects that did not undergo cognitive assessment were excluded from the analysis.

#### Significance Threshold and Multiple Comparisons Correction.

The significance threshold was set to *P* < .05 for all statistical tests. Correction for multiple hypotheses testing was performed in Python (statsmodels, v0.13.2)^79^ per group using the False Discovery Rate (FDR) procedure following Benjamini and Hochberg.^78^

## Results

An overview of the demographic and clinical characteristics of the final pooled samples for each clinical group is shown in table 1 and Supplementary table S1. For comparisons of these characteristics across patients and healthy controls, see Supplementary Results. For example, age and sex are comparable between patient groups and corresponding healthy control groups for each site.

### Consistently Lower Medial Nuclei Volumes Across All Stages of the Schizophrenia Spectrum, Lower Lateral Nuclei Volumes Only in Established Schizophrenia

**Fig. 2.**
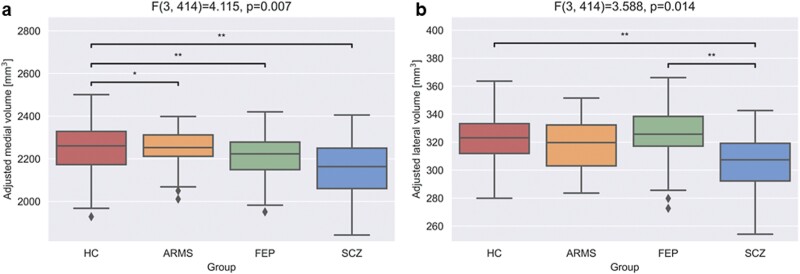
Volumetric differences in medial and lateral thalamic subregions between HC, ARMS subjects, individuals with FEP, and patients with established SCZ. Plotted volumes were adjusted for age, sex, and scanner-corrected eTIV. (a) Represents medial thalamic subregion volumes, (b) shows lateral subregion volumes. *P_unc_ < .05, **P_unc_ < .01 in post hoc analysis. ARMS, at-risk mental state; eTIV, estimated total intracranial volume; FEP, first-episode psychosis; HC, healthy controls; SCZ, schizophrenia.

To evaluate volumetric changes in grouped thalamic nuclei subregions across the schizophrenia spectrum, we applied one-way ANCOVA adjusting for age, sex, and eTIV followed by post hoc tests. We found group differences only in lateral and medial nuclei groups (figure 2, Supplementary table S3). More specifically, a significant group difference was detected in medial nuclei (*F*_3,414_ = 4.115, *P*_unc_ = .007, *P*_FDR_ = .042, η*p*^2^ = 0.029). Lower volumes were observed in ARMS (*P* = .046), FEP (*P* = .005), and established schizophrenia (*P* = .003) compared to the control group (figure 2a). Furthermore, we found a significant difference in the lateral nuclei subregion (*F*_3,414_ = 3.588, *P*_unc_ = .014, *P*_FDR_ = .042, η*p*^2^ = 0.026). Post hoc analysis demonstrated that volumes of lateral nuclei were significantly lower in schizophrenia patients compared to healthy controls (*P* = .005) as well as between schizophrenia patients and FEP (*P* = .010; figure 2b). These findings point to region-specific volume reductions in the medial and lateral thalamic nuclei, with the two nuclei showing a different pattern of reduction across the clinical groups.

#### Control Analyses

##### Group-based Volumetric Analysis of the Thalamic Subregions per Hemisphere

 To assess group differences in thalamic subregions per hemisphere, we conducted an ANCOVA adjusting for age, sex, and eTIV for each hemisphere separately. In line with our main analysis, we found that only lateral and medial subregions were significantly different between patients and controls (Supplementary table S4). For the medial nuclei, only the right hemisphere survived multiple comparisons correction (left: *F*_3,414_ = 2.903, *P*_unc_ = .035, *P*_FDR_ = .123, η*p*^2^ = 0.021; right: *F*_3,414_ = 4.342, *P*_unc_ = .005, *P*_FDR_ = .042, η*p*^2^ = 0.031). Post hoc tests show that volumes were lower in ARMS only in the right hemisphere (left: *P* = .107, right: *P* = .030), whereas medial nuclei for FEP (left: *P* = .030, right: *P* = .003) and SCZ (left: *P* = .009, right: *P* = .004) were lower for both hemispheres compared to controls. For the lateral nuclei, only the left hemisphere survived multiple comparisons correction (left: *F*_3,414_ = 4.091, *P*_unc_ = .007, *P*_FDR_ = .042, η*p*^2^ = 0.029; right: *F*_3,414_ = 2.780, *P*_unc_ = .041, *P*_FDR_ = .123, η*p*^2^ = 0.020). In accordance with the main analysis, only the schizophrenia group was affected (left: *P* = .010, right: *P* = .007).

##### Association of Thalamic Nuclei Volumes With Medication, Age, and Illness Duration

 Regarding medication status, approximately 70% of participants with FEP and almost all patients with schizophrenia were receiving antipsychotic medication (measured as CPZ equivalents) and/or medication with anticholinergic effect or burden (measured as ACB, see Supplementary table S1). To assess the link between medication status and thalamic nuclei volumes, multiple linear regression analyses were performed. Neither antipsychotic medication nor the anticholinergic burden of medication revealed a significant association with lateral or medial subregional volumes in any patient group investigated (Supplementary table S6).

##### Impact of Age or Illness Duration in the SCZ Group

 Furthermore, we investigated whether age or illness duration might impact thalamus nuclei volumes using multiple linear regression analyses in the schizophrenia group. Regarding lateral subregion volumes, no significant associations with age were found. In contrast, regarding medial subregion volumes, there was a significant link to age in the healthy control group (β-coefficient = −.451, *P* < .001) as well as the schizophrenia group (β-coefficient = −.373, *P* < .001; see Supplementary table S7). Since the groups are age-matched and age is related to medial volume in both groups, but medial volume is smaller in patients than in controls (even when controlling for age), we conclude that the aberration in medial volume is driven by one or more other factors, such as pathology or factors related to chronicity (eg, hospitalizations, years of antipsychotic medication intake, etc.). Moreover, there was no significant interaction between age and disease stage (lateral: β-coefficient = −.248, *P* = .131; medial: β-coefficient = −.138, *P* = .830), suggesting that the disorder effect on medial thalamus volume did not further increase with age (Supplementary table S8). Neither lateral nor medial subregion volumes were associated with illness duration in the schizophrenia group (Supplementary table S7.2), implying that there is no significant relationship between longer illness duration and greater reduction in thalamic nucleus volumes in schizophrenia.

### Lower Medial Nuclei Volumes are Specifically Linked to Cognitive Impairments

Next, we analyzed the relationship between altered medial and lateral thalamic nuclei subregions and different symptom clusters (ie, positive, negative, and cognitive) for each stage of the schizophrenia spectrum separately. We again want to stress here that clinical and cognitive data were only available for a subset of the participants and that neuropsychological test batteries differed between acquisition sites (see Supplementary table 1 for an overview). For each clinical group, we first analyzed cognitive impairments by examining differences in cognitive test performance compared to healthy controls using one-way ANCOVAs adjusting for age and sex. Second, multiple linear regression analyses were performed in a hierarchical framework to examine the associations between smaller volumes in the lateral or medial subregions and symptoms. For this purpose, we used four different models (see “Methods” section), each one adding a covariate to the model to track the influence of this covariate on the association between volume and symptoms.

**Fig. 3.**
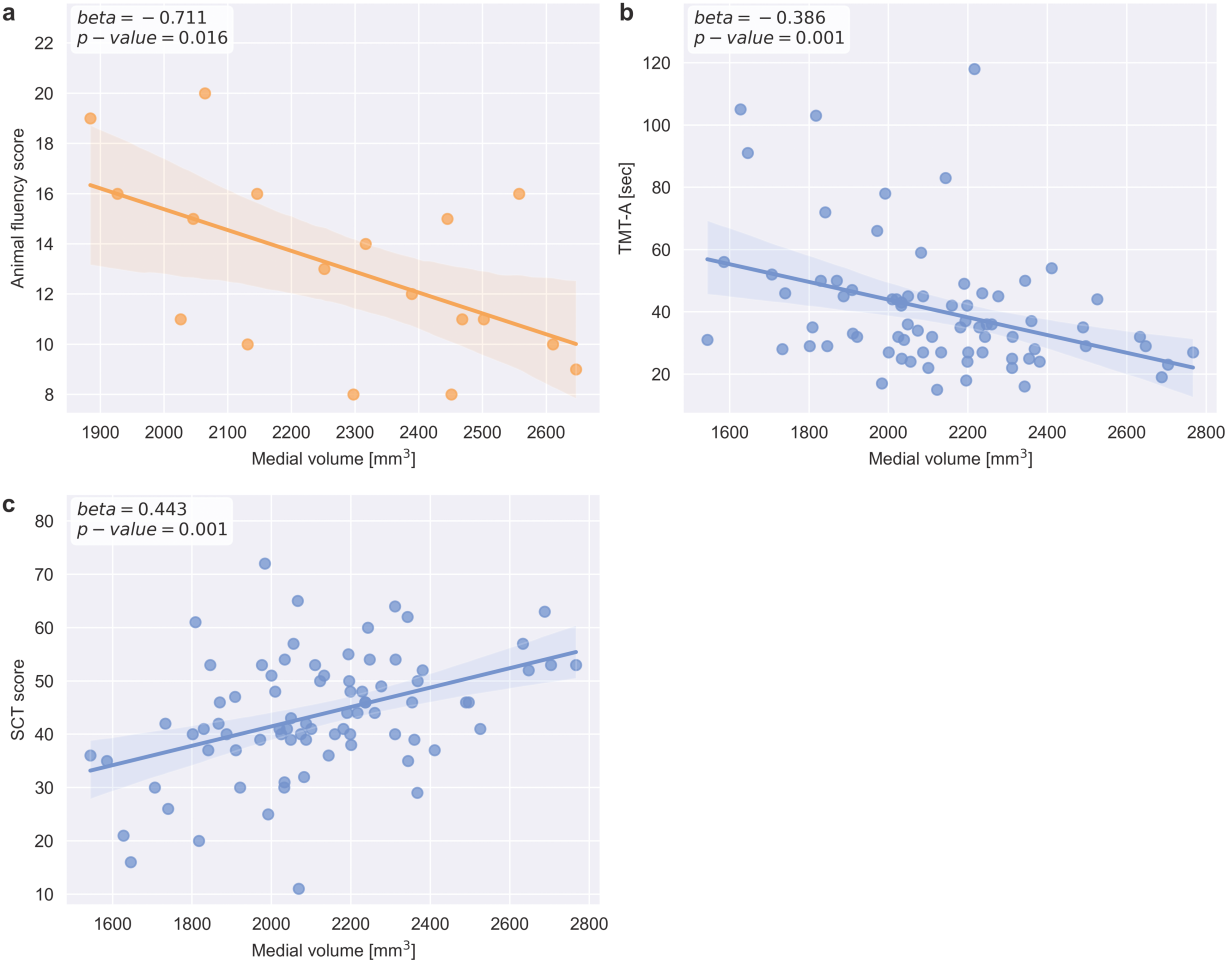
Association of medial thalamic nuclei volume with cognitive impairments across the schizophrenia spectrum. Volumes not adjusted for age, sex, and eTIV are shown for visualization purposes. (a) Significant association with animal fluency in at-risk mental state patients. (b) Significant association with TMT-A in established schizophrenia. (c) Significant association with SCT in established schizophrenia.

In ARMS individuals, we found significantly reduced animal fluency (*F*_1,81_ = 18.733, *P*_FDR_ < .001) but not phonemic fluency scores compared to healthy controls (Supplementary table S9.1). There was a significant anti-correlation between animal fluency and medial subregion volume in each of the four models (Supplementary table S10.1; model 1: β-coefficient = −.672, *P*_unc_ = .012; model 2: β-coefficient = −.674, *P*_unc_ = .018; model 3: β-coefficient = −.711, *P*_unc_ = .027; model 4: β-coefficient = −.677, *P*_unc_ = .028), that is, lower medial volume was associated with higher performance in the animal fluency task (Figure 3a). Negative and positive symptoms were not associated with lower medial nuclei volume.

In subjects with FEP, phonemic fluency was significantly lower compared to healthy controls (*F*_1,91_ = 14.351, *P*_FDR_ < .001; Supplementary table S9.2). There was no significant link between reductions in medial volume and phonemic fluency scores in any model (Supplementary table S10.2).

In established schizophrenia, cognitive performance has been tested not only with Verbal Fluency Tests but also with SCT and TMT-A in a larger number of patients (77 and 72 patients, respectively, out of 122 patients). We found that phonemic fluency was significantly reduced compared to healthy controls (*F*_1,150_ = 69.513, *P*_FDR_ < .001; Supplementary table S9.3). Furthermore, patients had significantly lower scores compared to healthy controls for SCT and TMT-A (SCT: *F*_1,143_ = 119.57, *P*_FDR_ < .001; TMT-A: *F*_1,138_ = 45.38, *P*_FDR_ < .001; Supplementary table S9.3). There was no association between medial nuclei volumes and phonemic fluency scores in any model (Supplementary table S10.3). Lower medial subregion volume was significantly anti-correlated with TMT-A scores in models 1, 2, and 3 (model 1: β-coefficient = −.425, *P* < .001; model 2: β-coefficient = −.425, *P* < .001; model 3: β-coefficient = −.386, *P* = .001; Figure 3b; Supplementary table S10.4), meaning that lower medial subregion volume is linked to worse TMT-A performance when correcting for the effect of sex and eTIV. The interaction was no longer significant when adding age into the model (model 4: β-coefficient = −.160, *P* = .127), suggesting that age influences the relationship between medial volume and TMT-A performance. Similarly, SCT scores were correlated with medial subregion volume changes in models 1, 2, and 3 (model 1: β-coefficient = .447, *P* < .001; model 2: β-coefficient = .446, *P* < .001; model 3: β-coefficient = .443, *P* < .001; Figure S3c; Supplementary table S10.5), also indicating higher cognitive symptom burden with lower thalamic nuclei volumes, but not in model 4 (model 4: β-coefficient = .156, *P* = .158), also indicating that age influences the association between medial volume and cognition impairment in SCZ.

Finally, cognitive impairments of patients with schizophrenia were not linked to lower lateral nuclei volumes (eg, TMT-A, model 3: β-coefficient = −.182, *P* = .153; see Supplementary tables S10.6–S10.8), implying that only medial nuclei volume decrease is implicated in cognitive impairments in the schizophrenia spectrum.

#### Specificity.

Of note, neither positive (assessed with PANSS positive or BPRS positive) nor negative symptoms (assessed with PANSS negative or SANS) were found to be significantly related to medial or lateral volume in any patient group (Supplementary table S10), suggesting that associations of medial nuclei volumes were specific for cognitive symptoms across stages of the spectrum.

In addition, there was no relation between medial subregional volume and any cognitive score detected in the healthy sample (Supplementary table S11). Yet, by the use of interaction analysis, we did not detect a specific link between medial nuclei volume and cognitive performance in the SCZ group (see Supplementary Results).

## Discussion

We examined thalamic nuclei volumes across the schizophrenia spectrum and associated nuclei volume changes with symptom scores. Medial nuclei volumes were specifically lower across the spectrum and associated with cognitive symptoms. In contrast, lateral nuclei volumes were lower only in established schizophrenia and were not linked to symptoms. To the best of our knowledge, this study is the first to demonstrate distinct volume reductions of thalamic nuclei across the entire schizophrenia spectrum. Regarding the potential course of schizophrenia, our findings might support a model of ongoing involvement of medial nuclei across developmental stages, impacting cognitive function, while thalamic impairment may encompass lateral nuclei at later stages.

### Thalamic Nuclei Show Different Patterns of Volume Reduction

#### Thalamic Volume Reductions are Specific to Medial and Lateral Nuclei.

The principal finding of the present study is that only medial and lateral nuclei in the schizophrenia spectrum had significantly lower volumes, while the volumes of the remaining nuclei did not differ significantly between groups. This implies a nuclei-specific impact of the disorder.

While postmortem findings regarding smaller thalamic nuclei in patients with schizophrenia are less consistent for the mediodorsal nucleus than the pulvinar,^23,24,36,37,74,80^ we note that neuroimaging literature has often reported alterations in both regions,^30,32^ but there are exceptions.^35,81^ The inconsistency between postmortem and imaging findings, particularly regarding the pulvinar, may be related to methodological differences and inherent limitations of each approach (eg, indirect measure of brain characteristics for MRI, small sample sizes for postmortem studies, etc.). Nevertheless, this discrepancy merits further investigation.

In contrast to the considerable literature in established schizophrenia, studies on pre-stages are sparse. However, there is evidence that the thalamus volume as a whole is smaller in FEP individuals,^82,83^ particularly the mediodorsal region,^84^ and that its volume is reduced longitudinally.^85^ Findings in ARMS have been controversial, with both reduced^86,87^ and increased^88^ volumes detected. So far, there is solely one investigation of thalamic nuclei volumes in ARMS, reporting no significant differences from healthy controls.^35^ However, only 4 of the 38 participants examined developed psychosis later, suggesting that these results may underestimate true differences.

#### Medial and Lateral Nuclei Reductions Exhibit Different Patterns Across the Schizophrenia Spectrum.

We noted distinct volume reduction patterns across disease stages. Specifically, lateral nuclei exhibited reductions solely in schizophrenia patients, whereas medial nuclei consistently showed lower volumes throughout the entire spectrum. As expected, medial volume reductions were less pronounced in ARMS and FEP subjects than in schizophrenia. Noteworthy, there was no significant difference of any subregional volume when assessing the Basel cohort only (see Supplementary Results, control analysis), before data harmonization. We argue that analysis in only one cohort is underpowered and that pooling data across sites is necessary to draw conclusions based on larger samples.

One interpretation of this pattern regarding schizophrenia development might be that medial nuclei impairments manifest early in the disease, possibly due to genetic predisposition, and worsen over disease stages. In contrast, lateral nuclei impairments may arise later during the disorder and result from different secondary mechanisms.

Indeed, there is evidence from genetic^89^ and animal^90,91^ studies that thalamic nuclei show different gene expression profiles and develop in distinct trajectories. We speculate that the alterations in the medial nuclei are due to differences in neurodevelopment, whereas this is less or not at all the case for the lateral nuclei. For instance, medial nuclei volumes were associated with a critical neurodevelopmental transcription factor,^89^ whose mutations have been linked to schizophrenia.^92,93^ This idea is supported by findings from neuroimaging studies. For instance, Xi et al^41^ compared cortico-thalamic functional connectivity within schizophrenia patients, their siblings, and healthy controls. Whereas increased sensorimotor-thalamic (ie, lateral) functional connectivity was specifically found in schizophrenia patients, decreased prefrontal-mediodorsal connectivity was shared between patients and their healthy siblings. Reduced prefrontal-mediodorsal connectivity was also observed in clinical high-risk individuals and in adolescents with early-onset schizophrenia,^15,40,42,94^ with subjects that later transitioned to schizophrenia showing the most apparent reductions.^42^ Together, the decrease of prefrontal-mediodorsal functional connectivity might to be at least partly genetically imprinted. Conversely, lower cell count might also lead to hypoconnectivity, although longitudinal studies are lacking. Our findings seem compatible with a developmental model of early impaired mediodorsal nuclei (ie, in ARMS) but later changes in lateral nuclei (ie, in an established disorder) in the course of schizophrenia. However, longitudinal studies are necessary to test this hypothesis.

### Volumetric Aberrations of the Medial Nuclear Group are Linked to Cognitive Impairments Across the Schizophrenia Spectrum

We detected significantly reduced cognitive performance in all clinical groups in at least one test of cognitive functioning. Furthermore, significant volume reductions in the medial nuclei group were specifically linked to cognitive symptoms in ARMS and SCZ patients. Thereby, specificity was apparent in two dimensions: (1) region-specific as medial not lateral volume reductions were associated with cognitive symptoms, and (2) symptom-specific as no link was detected between volume reductions and positive or negative symptoms.

In more detail, we found consistently lower cognitive scores in schizophrenia patients compared to controls for all cognitive tests, while we observed mixed findings in ARMS and FEP subjects (Supplementary table S9). This is in line with the general notion that cognitive symptoms are present in pre-stages but might differ regarding their domain and not be as severe and even more heterogeneous.^95–97^ Our results imply a worsening of symptoms across the spectrum and heterogeneity between subjects.

In the schizophrenia group, lower performances in TMT-A and SCT were significantly linked to lower medial nuclei volumes in several models, suggesting a key role of the medial thalamus in cognitive symptomatology. Medial volume is a significant predictor of cognition that is not explained by differences in gender or head size. However, when also accounting for age, cognition is not significantly explained by medial volume. As known from previous studies,^98–100^ age is strongly associated with volume and cognition. If variables in the model are highly correlated with each other (ie, multicollinearity), this leads to instability in estimates. Alternatively, the relationship between cognitive symptoms and volume might be non-linear, and age might capture these non-linear effects.

The implication of medial nuclei as a key modulator in cognitive functioning is well documented^101,102(pp. 312–317)^ and in line with its extensive reciprocal connections with medial and lateral prefrontal cortices (PFC), critically controlling cognitive functioning.^,101,102(p. 307)103^ Moreover, they may modulate the connectivity between the hippocampus and the mPFC, being also involved in the control of cognitive functions.^104,105^ Accordingly, lesion studies in patients with focal lesions demonstrated that the mediodorsal thalamus is necessary for cognitive control.^106,107^ Furthermore, abnormal prefrontal-mediodorsal functional connectivity and mediodorsal volume have been related to cognitive symptoms in patients with schizophrenia and FEP.^10,24,39–41^ However, this association between medial thalamus volume and cognitive impairment was not detected when age was additionally included as a covariate, pointing out that the association could be driven by age-related performance differences.

In contrast to the schizophrenia lesion model, where lower medial volumes are associated with reduced cognitive performance, we observed the opposite trend in ARMS. Specifically, we found a significant link between lower medial volume and higher cognitive scores. In general, this non-intuitive pattern might be attributed to a non-linear course of the relationship between medial thalamus volume and cognition over the course of schizophrenia. Given that prefrontal-mediodorsal connectivity seems to be neurodevelopmentally decreased in schizophrenia^41^ and that dysconnectivity might entail^108,109^ or is at least related to^110^ gray matter loss, prefrontal-mediodorsal connectivity going along with cognitive decline might precede substantial volume reductions in the medial thalamus across the whole at-risk population. The anti-correlation observed in ARMS may suggest that compensatory mechanisms are effective early in the disease course leading to a relatively better performance in cognitive performance despite thalamic structural differences. However, as the disease progresses to FEP and SCZ stages, these compensatory mechanisms might become less efficient, resulting in no significant correlation and, ultimately, in a different pattern of correlation.

In healthy controls, we did not find a significant correlation between cognition and medial thalamus volume. This is in contrast to a previous study,^30^ but might be explained by the employed cognitive testing, mainly focused on processing speed-related cognitive functions. Nevertheless, our results show a significant link between cognitive symptomatology and medial thalamic volume reductions in some spectrum groups, following the assumption that especially medial nuclei are relevant for various domains of cognition.^19,101,106^

### Strengths and Limitations

This study extends prior research in several ways: first, the pipeline utilized to segment thalamic nuclei is well evaluated^33^ and has been successfully applied to a variety of disorders.^30,31,69,111^ Therefore, a well-justified and high-resolution analysis of thalamic subregions is provided. Second, intensive quality control as well as adjustment for multi-site batch effects attest to the methodological precision of this investigation. Third, the presentation of findings based on a large sample encompassing multiple stages of the schizophrenia spectrum allows the generation of valuable hypotheses regarding developmental trajectories.

Despite these strengths, several limitations require consideration. Segmentation of thalamic nuclei suffers from poor contrast of thalamic margins on T1w images. Although we intensively scrutinized segmentation quality according to the latest recommendations^69,70^ and have excluded some nuclei due to their small size and poor contrast in 1 mm^3^ T1w images, we are aware that criticism exists regarding the anatomical relevance of this pipeline.^112^ Furthermore, despite correcting for batch effects of MRI data using gold standard procedures,^71^ some effects resulting from site and hardware differences may remain. Especially, since the results for the Basel site alone were not significant for schizophrenia pre-stages, we cannot rule out that effects were affected by the harmonization procedure. By demonstrating that these differences are not significant, large effects are not to be anticipated. Additionally, due to the lack of respective tools, clinical and cognitive data were not harmonized across sites, possibly leading to non-biological sources of variance in the data. Although we emphasize the uniqueness and importance of the spectrum design of our study for hypotheses regarding disease progression, we caution that a cross-sectional design does not permit temporal inference of thalamic nuclei volume alterations. In addition, comparisons between the data sets regarding clinical and cognitive characteristics were only possible to a limited extent due to the different batteries employed, which were limited in scope (ie, mainly assessing processing speed) and were only completed by a subset of the participants.

## Supplementary Material

Supplementary material is available at https://academic.oup.com/schizophreniabulletin/.

sbae037_suppl_Supplementary_Material
