# Supplementary material for: Distinct Volume Alterations of Thalamic Nuclei Across the Schizophrenia Spectrum
Source: Schizophr Bull. 2024 Apr 5;50(5):1208–22. doi: 10.1093/schbul/sbae037 (PMC11349018; doi:10.1093/schbul/sbae037)
Supplement: sbae037_suppl_Supplementary_Material [file sbae037_suppl_supplementary_material.docx]

**Supplementary Material** for:

Distinct volume alterations of thalamic nuclei across the schizophrenia spectrum

By Thalhammer et. al.

# Table of contents

[Table of contents 2](#_Toc154065442)

[Supplementary methods 4](#_Toc154065443)

[MRI data acquisition 4](#_Toc154065444)

[Quality assessment of cortical reconstruction 4](#_Toc154065445)

[Quality assessment of the thalamus segmentation 5](#_Toc154065446)

[Assessment of scanner harmonization effect 6](#_Toc154065447)

[Statistical analysis of demographics 6](#_Toc154065448)

[Control analyses for group difference in thalamic subregion volumes 6](#_Toc154065449)

[Associations between thalamic subregion volumes and medication 6](#_Toc154065450)

[Association between thalamus subregional volumes, age, and illness duration 6](#_Toc154065451)

[Control analysis for association of medial subregion volume with cognitive performance – analysis of cognition*group interaction 7](#_Toc154065452)

[Supplementary results 9](#_Toc154065453)

[Demographics 9](#_Toc154065454)

[Control analysis: group-based volumetric analysis of the thalamic subregions in the Basel cohort 9](#_Toc154065455)

[Control analysis for association of medial subregion volume with cognitive performance – analysis of cognition*group interaction 9](#_Toc154065456)

[Supplementary tables 11](#_Toc154065457)

[Table S1: Overview of the clinical characteristics and scores for positive, negative, and cognitive symptoms 11](#_Toc154065458)

[Table S2: Overview of relevant cognitive processes associated with cognitive tests conducted in our study as well as linked thalamic outcomes 14](#_Toc154065459)

[Table S3: Group-based volumetric analysis of the thalamic subregions 15](#_Toc154065460)

[Table S4: Group-based volumetric analysis of the thalamic subregions per hemisphere 16](#_Toc154065461)

[Table S5: Group-based volumetric analysis of the thalamic subregions in the Basel cohort 17](#_Toc154065462)

[Table S6: Association of regional thalamic nuclei volumes with medication 18](#_Toc154065463)

[Table S7: Association of regional thalamic nuclei volume with age and illness duration 19](#_Toc154065464)

[Table S8: Moderation analysis of age and group 20](#_Toc154065465)

[Table S9: Group differences in cognition 21](#_Toc154065466)

[Table S10: Hierarchical multiple linear regression analysis to assess the link between positive, negative, and cognitive symptoms with thalamic nuclei volumes 22](#_Toc154065467)

[S10.1: Association between medial nuclei volume and animal fluency in ARMS 22](#_Toc154065468)

[S10.2: Association between medial nuclei volume and phonemic fluency in FEP 24](#_Toc154065469)

[S10.3: Association between medial nuclei volume and phonemic fluency in SCZ 26](#_Toc154065470)

[S10.4: Association between medial nuclei volume and TMT-A in SCZ 28](#_Toc154065471)

[S10.5: Association between medial nuclei volume and SCT in SCZ 30](#_Toc154065472)

[S10.6: Association between lateral nuclei volume and phonemic fluency in SCZ 32](#_Toc154065473)

[S10.7: Association between lateral nuclei volume and TMT-A in SCZ 34](#_Toc154065474)

[S10.8: Association between lateral nuclei volume and SCT in SCZ 36](#_Toc154065475)

[Table S11: Control analysis: hierarchical multiple linear regression analysis of cognitive impairments and thalamic nuclei volumes in healthy controls 38](#_Toc154065476)

[Supplementary figures 39](#_Toc154065477)

[References 40](#_Toc154065478)

# Supplementary methods

## MRI data acquisition

*Munich dataset.* Data were acquired on a hybrid whole-body 3 T MR Biograph PET/MRI scanner (Siemens-Healthineers, Erlangen, Germany) at the Klinikum rechts der Isar, Munich, Germany. A vendor-supplied 12-channel phase-array coil was used. T_1_-weighted MPRAGE sequence parameters were: TR/TE/flip angle: 2300 ms/2.98 ms/9°; 160 slices (gap 0.5 mm) covering the whole brain; FoV: 256 mm; matrix size: 256×256; voxel size: 1×1×1mm^3^.

*COBRE dataset.* All participants were scanned in a 3 T Siemens Trio MR scanner with a 12-channel radio frequency coil. A T_1_-weighted MPRAGE sequence was collected using the following parameters: TR/TE/flip angle: 2530/(1.64, 3.5, 5.36, 7.22, 9.08)/7°; FoV: 256 mm; matrix size: 256×256; voxel size: 1×1×1mm^3^. Consult <http://fcon_1000.projects.nitrc.org/indi/retro/cobre.html> for more information.

*Zurich dataset*. The MR-Zentrum of the Psychiatric Hospital, University of Zurich collected data on a Philips Achieva 3 T MR scanner with a 32-channel SENSE head coil. Anatomical images were recorded using an ultra-fast gradient echo using an ultra-fast gradient echo T_1_-weighted sequence with TR/TE/flip angle: 8.4 ms/3.8 ms/8°; 160 slices covering the whole brain; FoV: 256 mm; matrix size: 240×240; voxel size: 1×1×1mm^3^.

*Basel dataset*: Data were collected on a 3 T MR scanner (Magnetom Verio, Siemens Healthcare, Erlangen, Germany) at the Basel University Hospital. A T_1_-weighted MPRAGE sequence was acquired using TR/TE/flip angle: 2000 ms/3.4 ms/8°; 160 slices covering the whole brain; FoV: 176 mm; matrix size: 256×256; voxel size: 1×1×1mm^3^.

## Quality assessment of cortical reconstruction

To assess reconstruction quality, the FreeSurfer QAtools pipeline for version 7+ was applied (<https://surfer.nmr.mgh.harvard.edu/fswiki/QATools>), which generates lateral and medial snapshots of the reconstruction for visual inspection. Assessment was then based on the guidelines provided by the ENIGMA Working Group (<https://enigma.ini.usc.edu/protocols/imaging-protocols/>). 25 subjects were excluded due to insufficient reconstruction quality.

## Quality assessment of the thalamus segmentation

Quality assessment of the thalamus segmentation was conducted following a recent thalamus nuclei analysis on the subregional level in obsessive-compulsive disorder^1^ and recommendations by the ENIGMA consortium.^2^ Due to general poor segmentation results, the lateral and medial geniculate nuclei were excluded from further analyses. During the visual quality inspection of the thalamus segmentation, some nuclei showed the strongest deviations from the recon-all aseg surfaces (e.g., overlap with white matter mask or surfaces of other structures, e.g., the pallidum), especially the ventral posterolateral (VPL) and ventral lateral posterior (VLp). Thus, these nuclei were also excluded from further analyses. The limitans suprageniculate was also excluded due to its small size and the resulting vulnerability for inaccurate segmentation. Moreover, statistical outliers were calculated for each of the remaining 22 nuclei per hemisphere (below Q1 – 1.5 * IQR or above Q3 + 1.5 * IQR) per site. 17 subjects with more than 10 outliers were excluded. Segmentation quality of these remaining outliers was visually inspected in Freeview. For 24 subjects, we saw a large overlap of segmented nuclei with the surrounding gray matter such as the pallidum or putamen when loading the thalamus segmentation and pre-processed T1w image in Freeview. For one subject, nuclei overlapped with the lateral ventricles. For one subject, the thalamus surface was not segmented as smoothly as to be expected from its anatomy. As an additional quality measure, thalamus segmentation that deviated too much (i.e., more than 15 %) from the native (aseg) FreeSurfer segmentation of the thalamus were visually inspected. For five subjects, there was a large overlap with surrounding white matter. In total, additional 48 subjects were excluded from further analyses.

## Assessment of scanner harmonization effect

To assess the effect of scanner harmonization, an ANCOVA with subregional volume as dependent variable and age, sex, and eTIV as covariates was performed between sites using Pyton’s pingouin package (v 0.5.2).^3^ Results are visualized in Figure S1.

## Statistical analysis of demographics

Demographical aspects were analyzed using custom-made scripts in Python 3.9.7. Analysis of Variance (ANOVA) from the pingouin package was applied to assess age difference, whereas a Χ^2^-test (*chi2_contingency* from scipy, v 1.7.0)^4^ was used to compare gender across groups.

## Control analyses for group difference in thalamic subregion volumes

### Associations between thalamic subregion volumes and medication

Testing the associations between medication and thalamic subregion volumes was conducted in SPSS using multiple linear regression analyses. Thereby, the respective significantly different subregion per group was described using the following formula:

$$volume= \beta_{0}+\beta_{1}*eTIV+\beta_{2}*sex+\beta_{3}*CPZ+\beta_{4}+ACB$$

### Association between thalamus subregional volumes, age, and illness duration

Another multiple linear regression model was used to determine the association between age or illness duration and subregional volumes in all participant groups. For assessing the effect of age, the following formula was used:

$$volume= \beta_{0}+\beta_{1}*eTIV+\beta_{2}*sex+\beta_{3}*age$$

For assessing the effect of illness duration on thalamic nuclei volume, duration was added to formula above:

$$volume= \beta_{0}+\beta_{1}*eTIV+\beta_{2}*sex+\beta_{3}*age+ \beta_{4}*duration$$

Next, a moderation analysis was performed using the recommendations by Hayes^5^ implemented in the PROCESS package (v 4.2; <https://www.processmacro.org/download.html>) for SPSS Statistics.

To analyze the interaction between age and diagnosis, diagnosis was entered as the independent variable, age as a moderator variable, and the subregional volume as the dependent variable. Sex and eTIV were entered as covariates. Continuous variables were mean centered. In summary, the following regression model was assessed by the toolbox:

$$volume= \beta_{0}+\beta_{1}*\left( age-{age}_{mean} \right)+\beta_{2}*diagnosis+\beta_{3}*\left( age-{age}_{mean} \right)*diagnosis+ \beta_{4}*sex+\beta_{5}*\left( eTIV-{eTIV}_{mean} \right)$$

To analyze the interaction between age and illness duration in schizophrenia patients, illness duration was entered as the independent variable, age as a moderator variable, and subregional volume as the dependent variable. Again, sex, and eTIV were entered as covariates.

## Control analysis for association of medial subregion volume with cognitive performance – analysis of cognition*group interaction

*To assess whether the relation between medial nuclei volume and cognitive symptomatology is specific to the schizophrenia spectrum, we compared the regression coefficients between thalamic subregion volume and cognitive scores by adding an interaction term between cognition and group to the regression analysis. Age, sex, and estimated total intracranial volume (eTIV) were added as covariates.*

$$volume=\beta_{0}+\beta_{1}*eTIV+\beta_{2}*sex+\beta_{3}*age+\beta_{4}*cognition+\beta_{5}*group$$

$$+ \beta_{5}*cognition*group$$

*Since there was no consistent cognitive score available across the whole spectrum, we analyzed the interactions between the SCZ and HC groups and TMT-A, SCT, and phonemic fluency, as well as between FEP/ARMS and HC groups and phonemic fluency.*

# Supplementary results

## Demographics

Due to the characteristics of the disorder, age significantly differs between groups in the overall sample (F_3.414_=32.21, p<0.001). However, per site, controls match in age with patients, except for the Zurich cohort (Basel: F_2,167_=2.953, p=0.055; COBRE: F_1,113_=0.410, p=0.523; Munich: F_1,41_=1.094, p=0.302; Zurich: F_2,87_=9.020, p<0.001). Similarly, biological sex also significantly varied between groups across sites (Χ^2^_3_=10.436, p=0.015) due to different study designs. This difference was also evident when assessing the Basel (Χ^2^_2_=12.31, p=0.002), but not the COBRE (Χ^2^_1_=0.735, p=0.391), Munich (Χ^2^_1_<0.001, p=0.998), and Zurich (Χ^2^_2_=1.313, p=0.519) studies separately.

## Control analysis: group-based volumetric analysis of the thalamic subregions in the Basel cohort

Since limitations of scanner harmonization procedures exist,^6,7^ we analyzed group differences in thalamic nuclei subregions separately for the Basel cohort, which was the only site where ARMS subjects were recruited. In order to test group differences within the Basel cohort (composed of 38 HC, 66 ARMS, and 66 FEP subjects), we conducted a one-way ANCOVA adjusting for age, sex, and eTIV on unharmonized data. There was no significant difference between groups for any nucleus (Supplementary Table S5).

## Control analysis for association of medial subregion volume with cognitive performance – analysis of cognition*group interaction

To determine whether the association between medial thalamic nuclei volumes and cognitive scores were specific to the schizophrenia spectrum, we included a cognition-by-group interaction term into our multiple linear regression model framework for the medial subregion (model 4, i.e., adding sex, eTIV; and age as covariates). For schizophrenia and healthy controls, there was no significant TMT-A-by-group (β-coefficient=-0.573, p=0.071), SCT-by-group (β-coefficient=0.409, p=0.118), or phonemic fluency-by-group interaction (β-coefficient=-0.229, p=0.240). For FEP and healthy controls, there was no significant phonemic fluency-by-group interaction (β-coefficient=-0.323, p_unc_=0.258). In contrast, there was a significant animal fluency-by-group interaction for ARMS and HC (β-coefficient=-0.851, p_unc_=0.005). Since there was a significant interaction between cognition and group only in ARMS and a significant relationship between cognition and medial volume was only found in ARMS and SCZ groups, we argue that there is a cognition-thalamic nuclei volume link for some spectrum groups.

# Supplementary tables

## Table S1: Overview of the clinical characteristics and scores for positive, negative, and cognitive symptoms

Table S1: Overview of the clinical characteristics and scores for positive, negative, and cognitive symptomatology available. Data are listed for the final data sample after exclusion due to quality control. Number of participants data are available for is denoted as n. As acquisition sites’ assessments differed, the sites that included the scale in their protocols are listed under ‘Sites’.

| **Variable** | **Sites** | **Measure** | **HC**  **(n=137)** | **ARMS**  **(n=66)** | **FEP**  **(n=89)** | **SCZ**  **(n=126)** |
| --- | --- | --- | --- | --- | --- | --- |
| **Age** [years] | B, C, M, Z | mean (SD) | 32.93 (10.88) | 25.02 (5.64) | 26.67 (7.11) | 37.52 (12.17) |
| **Sex** | B, C, M, Z | n (%) females | 58 (42) | 18 (27) | 23 (26) | 34 (27) |
| **Illness duration** [years] | B, C, M, Z | range |  |  |  | 1 - 41 |
|  |  | mean (SD) |  |  |  | 13.67 (10.12) |
| **Medication** |  |  |  |  |  |  |
| CPZ | B, C, M, Z | n data available |  |  | 84 | 121 |
|  |  | n medicated [%] |  |  | 59 (70.24) | 115 (95.04) |
|  |  | mean (SD) [mg/d] |  |  | 203.87 (250.04) | 467.72 (393.49) |
| ACB | B, C, M, Z | n data available |  |  | 88 | 122 |
|  |  | n medicated [%] |  |  | 64 (72.73) | 118 (96.72) |
|  |  | mean (SD) [a.u.] |  |  | 1.55 (1.43) | 2.78 (1.62) |

Table S1 (continued)

| **Positive symptoms** |  |  |  |  |  |  |
| --- | --- | --- | --- | --- | --- | --- |
| PANSS positive | C, M, Z | n,  mean (SD) |  |  | n=23  6.17 (1.80) | n=121  11.65 (5.33) |
| BPRS positive | B | n,  mean (SD) |  | n=58  6.47 (2.35) | n=44  7.83 (3.04) |  |
| **Negative symptoms** |  |  |  |  |  |  |
| PANSS negative | C, M, Z | n  mean (SD) |  |  |  | n=121  14.28 (5.43) |
| BPRS negative | B | n  mean (SD) |  | n=58  7.93 (2.45) | n=43  14.74 (4.79) |  |
| SANS | B | n  mean (SD) |  | n=56  17.15 (13.23) | n=43  23.26 (17.26) |  |

Table S1 (continued)

| **Cognitive performance** |  |  |  |  |  |  |
| --- | --- | --- | --- | --- | --- | --- |
| Animal fluency | B, Z | n  mean (SD) | n=67  23.34 (12.15) | n=18  13.00 (3.58) | n=35  27.57 (8.64) | n=87  22.86 (8.34) |
| Phonemic fluency | B, Z | n  mean (SD) | n=67  25.28 (5.83) | n=18  22.28 (6.38) | n=35  19.17 (7.30) | n=87  15.57 (7.92) |
| TMT-A [sec] | C, M | n  mean (SD) | n=67  23.57 (7.68) |  |  | n=72  39.29 (18.62) |
| SCT | C, M | n  mean (SD) | n=67  62.03 (10.80) |  |  | n=77  43.63 (11.38) |

*a.u. arbitrary units, ACB anti-cholinergic burden, ARMS at-risk mental state, B Basel, BPRS Brief Psychiatric Rating Scale, C COBRE, CPZ chlorpromazine equivalents, d day, FEP first-episode psychosis, HC healthy control, M Munich, mg milligrams, PANSS Positive and Negative Syndrome Scale, SANS Scale for the Assessment of Negative Symptoms, SCT Symbol Coding Task of the Brief Assessment of Cognition in Schizophrenia, SCZ schizophrenia, SD standard deviation, sec seconds, TMT-A Trail Making Test Part A, Z Zurich.*

## Table S2: Overview of relevant cognitive processes associated with cognitive tests conducted in our study as well as linked thalamic outcomes

Table S2: Overview of relevant cognitive processes associated with cognitive tests conducted in our study as well as linked thalamic outcomes.

| ***Cognitive Test*** | ***Relevant cognitive processes*** | ***Associated thalamic outcomes*** |
| --- | --- | --- |
| ***Verbal Fluency*** | *(auditory) attention, verbal long-term memory, mental flexibility, ability to inhibit responses, processing speed^8-10^* | - *Thalamus is involved in long-term memory, especially anterior and mediodorsal nuclei^11^* - *mediodorsal thalamus implicated in behavioral flexibility^12^* |
| ***TMT-A*** | *processing speed and visual attention^13^, psychomotor speed, visuospatial search and target-directed motor tracking^14^* | - *TMT performance lower in patients with lesions involved in mediodorsal thalamus^15^* |
| ***SCT*** | *processing speed, attention^16^* | - *Lower thalamic volume in multiple sclerosis patients is connected to lower processing speed and attention as measured via a symbol digit modality test^17,18^* - *Lower ventral nuclei volume in schizophrenia patients compared to controls was associated with lower scores in Brief Assessment of Cognition in Schizophrenia (BACS, i.e., test battery, SCT is part of)^19^* |

*SCT* *Symbol Coding Task of the Brief Assessment of Cognition in Schizophrenia, TMT-A Trail Making Test Part A.*

## Table S3: Group-based volumetric analysis of the thalamic subregions

Table S3: Group-based volumetric analysis of the thalamic subregions. A one-way ANCOVA adjusted for age, sex, and eTIV was conducted.

| **Region** | **F_3,414_** | **p-value** | **p_FDR_** | **Significance** |
| --- | --- | --- | --- | --- |
| **Anterior** | 1.075 | 0.360 | 0.432 |  |
| **Lateral** | 3.588 | 0.014 | 0.042 | * |
| **Ventral** | 0.750 | 0.523 | 0.523 |  |
| **Intralaminar** | 2.190 | 0.089 | 0.178 |  |
| **Medial** | 4.115 | 0.007 | 0.042 | * |
| **Pulvinar** | 1.075 | 0.359 | 0.432 |  |

*ANCOVA Analysis of Covariance, eTIV estimated total intracranial volume, FDR False Discovery Rate.*

## Table S4: Group-based volumetric analysis of the thalamic subregions per hemisphere

Table S4: Group-based volumetric analysis of the thalamic subregions. A one-way ANCOVA adjusted for age, sex, and eTIV was conducted.

| **Region** | **Hemisphere** | **F_3,411_** | **p-value** | **p_FDR_** | **Significance** |
| --- | --- | --- | --- | --- | --- |
| **Anterior** | L | 2.088 | 0.101 | 0.173 |  |
|  | R | 0.449 | 0.718 | 0.718 |  |
| **Lateral** | L | 4.091 | 0.007 | 0.042 | * |
|  | R | 2.780 | 0.041 | 0.123 |  |
| **Ventral** | L | 1.767 | 0.153 | 0.230 |  |
|  | R | 0.824 | 0.481 | 0.577 |  |
| **Intralaminar** | L | 2.355 | 0.071 | 0.142 |  |
|  | R | 2.567 | 0.054 | 0.130 |  |
| **Medial** | L | 2.903 | 0.035 | 0.123 |  |
|  | R | 4.342 | 0.005 | 0.042 | * |
| **Pulvinar** | L | 1.344 | 0.260 | 0.347 |  |
|  | R | 0.508 | 0.677 | 0.718 |  |

*ANCOVA Analysis of Covariance, eTIV estimated total intracranial volume, FDR False Discovery Rate.*

## Table S5: Group-based volumetric analysis of the thalamic subregions in the Basel cohort

*Table S5: Group-based volumetric analysis of the thalamic subregions for Basel dataset. A one-way ANCOVA adjusted for age, sex, and eTIV was conducted. The Basel cohort includes 38 HC, 66 ARMS, and 66 FEP individuals.*

| **Region** | **F_2,164_** | **p-value** | **p_FDR_** | **Significance** |
| --- | --- | --- | --- | --- |
| **Anterior** | 0.525 | 0.592 |  |  |
| **Lateral** | 0.919 | 0.401 |  |  |
| **Ventral** | 1.095 | 0.337 |  |  |
| **Intralaminar** | 2.413 | 0.093 |  |  |
| **Medial** | 1.966 | 0.143 |  |  |
| **Pulvinar** | 1.290 | 0.278 |  |  |

*ANCOVA Analysis of Covariance, eTIV estimated total intracranial volume, FDR False Discovery Rate.*

## Table S6: Association of regional thalamic nuclei volumes with medication

Table S6.1: Association of regional thalamic nuclei volume with medication in patients with first-episode psychosis.

| **Region** | **Regressor** | **β-coefficient** | **p-value** | **Significance** |
| --- | --- | --- | --- | --- |
| **Medial** | CPZ | -0.156 | 0.217 |  |
|  | ACB | 0.049 | 0.699 |  |

*ACB anticholinergic burden of medication (applied units), CPZ chlorpromazine equivalents (mg/d), d day, FDR false discovery rate, mg milligrams.*

Table S6.2: Association of regional thalamic nuclei volume with medication in patients with schizophrenia.

| **Region** | **Regressor** | **β-coefficient** | **p-value** | **Significance** |
| --- | --- | --- | --- | --- |
| **Medial** | CPZ | 0.004 | 0.968 |  |
|  | ACB | -0.141 | 0.143 |  |
| **Lateral** | CPZ | -0.018 | 0.858 |  |
|  | ACB | -0.108 | 0.273 |  |

*ACB anticholinergic burden of medication (applied units), CPZ chlorpromazine equivalents (mg/d), d day, FDR false discovery rate, mg milligrams.*

## Table S7: Association of regional thalamic nuclei volume with age and illness duration

Table S7.1: Association of regional thalamic nuclei volume with age in patients with schizophrenia.

| **Region** | **Regressor** | **β-coefficient** | **p-value** | **Significance** |
| --- | --- | --- | --- | --- |
| **Medial** | Age | -0.484 | <0.001 | *** |
| **Lateral** | Age | -0.262 | 0.002 | ** |

Table S7.2: Association of regional thalamic nuclei volume with illness duration in patients with schizophrenia while additionally correcting for age.

| **Region** | **Regressor** | **β-coefficient** | **p-value** | **Significance** |
| --- | --- | --- | --- | --- |
| **Medial** | Age | -0.373 | <0.001 | *** |
|  | Duration | -0.168 | 0.083 |  |
| **Lateral** | Age | -0.169 | 0.125 |  |
|  | Duration | -0.136 | 0.218 |  |

Table S7.3: Association of regional thalamic nuclei volume with age in healthy controls.

| **Region** | **Regressor** | **β-coefficient** | **p-value** | **Significance** |
| --- | --- | --- | --- | --- |
| **Medial** | Age | -0.451 | <0.001 | *** |
| **Lateral** | Age | -0.112 | 0.149 |  |

## Table S8: Moderation analysis of age and group

Table S8: Results of moderation analysis testing the interaction between group and age in patients with schizophrenia.

| **Region** | **Regressor** | **β-coefficient** | **T-statistic** | **SE** | **p-value** | **Significance** |
| --- | --- | --- | --- | --- | --- | --- |
| **Medial** | Age*group | -0.138 | -0.215 | 0.642 | 0.830 |  |
| **Lateral** | Age*group | -0.248 | -1.514 | 0.167 | 0.131 |  |

*SE standard error.*

## Table S9: Group differences in cognition

Table S9.1: Group differences between HC and ARMS assessed with ANCOVA using age and sex as covariates. The number of subjects for which cognitive data were available is noted as n_ARMS_ and n_HC,_ respectively.

|  | **F_1,81_** | **p-value** | **n_ARMS_** | **n_HC_** | **Significance** |
| --- | --- | --- | --- | --- | --- |
| Animal fluency | 18.733 | <0.001 | 18/66 | 67/136 | *** |
| Phonemic fluency | 0.954 | 0.332 | 18/66 | 67/136 |  |

*ANCOVA analysis of covariance, ARMS at-risk mental state, FDR False Discovery Rate, HC healthy controls.*

Table S9.2: Group differences between HC and FEP assessed with ANCOVA using age and sex as covariates. The number of subjects for which cognitive data were available is noted as n_FEP_ and n_HC,_ respectively.

|  | **F_1,98_** | **p-value** | **n_FEP_** | **n_HC_** | **Significance** |
| --- | --- | --- | --- | --- | --- |
| Animal fluency | 0.909 | 0.343 | 35/89 | 67/136 |  |
| Phonemic fluency | 14.351 | <0.001 | 35/89 | 67/136 | *** |

*ANCOVA analysis of covariance, FDR False Discovery Rate, FEP first-episode psychosis, HC healthy controls.*

Table S9.3: Group differences between HC and SCZ assessed with ANCOVA using age and sex as covariates. The number of subjects for which cognitive data were available is noted as n_SCZ_ and n_HC,_ respectively.

|  | **F_1,150_** | **p-value** | **n_SCZ_** | **n_HC_** | **Significance** |
| --- | --- | --- | --- | --- | --- |
| Animal fluency | 0.012 | 0.912 | 87/123 | 67/136 |  |
| Phonemic fluency | 69.513 | <0.001 | 87/123 | 67/136 | *** |
| SCT | 129.570 | <0.001 | 77/123 | 67/136 | *** |
| TMT-A | 45.382 | <0.001 | 72/123 | 67/136 | *** |

*ANCOVA analysis of covariance, HC healthy controls,* *SCT* *Symbol Coding Task of the Brief Assessment of Cognition in Schizophrenia, SCZ schizophrenia,* *TMT-A Trail Making Test Part A.*

## Table S10: Hierarchical multiple linear regression analysis to assess the link between positive, negative, and cognitive symptoms with thalamic nuclei volumes

### S10.1: Association between medial nuclei volume and animal fluency in ARMS

Table S10.1.1: Association between medial nuclei volume and cognitive performance measured with animal fluency when not accounting for sex, eTIV, or age in ARMS subjects (hierarchy level 1).

| **Region** | **Regressor** | **β-coefficient** | **p-value** | **Significance** |
| --- | --- | --- | --- | --- |
| **Medial** | BPRS p | -0.439 | 0.261 |  |
|  | SANS | 0.181 | 0.630 |  |
|  | Animal fluency | -0.672 | 0.012 | * |

*ARMS at-risk mental state, BPRS Brief Psychiatric Rating Scale, p positive, SANS Scale for the Assessment of Negative Symptoms.*

Table S10.1.2: Association between medial nuclei volume and cognitive performance when accounting for sex in ARMS subjects (hierarchy level 2).

| **Region** | **Regressor** | **β-coefficient** | **p-value** | **Significance** |
| --- | --- | --- | --- | --- |
| **Medial** | BPRS p | -0.440 | 0.281 |  |
|  | SANS | 0.183 | 0.646 |  |
|  | Animal fluency | -0.674 | 0.018 | * |
|  | Sex | 0.006 | 0.979 |  |

*ARMS at-risk mental state, BPRS Brief Psychiatric Rating Scale, p positive, SANS Scale for the Assessment of Negative Symptoms.*

Table S10.1.3: Association between medial nuclei volume and cognitive performance when accounting for sex and eTIV in ARMS subjects (hierarchy level 3).

| **Region** | **Regressor** | **β-coefficient** | **p-value** | **Significance** |
| --- | --- | --- | --- | --- |
| **Medial** | BPRS p | -0.363 | 0.384 |  |
|  | SANS | 0.136 | 0.736 |  |
|  | Animal fluency | -0.711 | 0.016 | * |
|  | Sex | 0.334 | 0.437 |  |
|  | eTIV | 0.392 | 0.366 |  |

*ARMS at-risk mental state, BPRS Brief Psychiatric Rating Scale, eTIV estimated total intracranial volume, p positive, SANS Scale for the Assessment of Negative Symptoms.*

Table S10.1.4: Association between medial nuclei volume and cognitive performance when accounting for sex, eTIV, and age in ARMS subjects (model 4).

| **Region** | **Regressor** | **β-coefficient** | **p-value** | **Significance** |
| --- | --- | --- | --- | --- |
| **Medial** | BPRS p | -0.391 | 0.367 |  |
|  | SANS | 0.124 | 0.766 |  |
|  | Animal fluency | -0.677 | 0.028 | * |
|  | Sex | 0.615 | 0.551 |  |
|  | eTIV | 0.342 | 0.450 |  |
|  | Age | -0.143 | 0.573 |  |

*ARMS at-risk mental state, BPRS Brief Psychiatric Rating Scale, eTIV estimated total intracranial volume, p positive, SANS Scale for the Assessment of Negative Symptoms.*

### S10.2: Association between medial nuclei volume and phonemic fluency in FEP

Table S10.2.1: Association between medial nuclei volume and cognitive performance measured with phonemic fluency when not accounting for sex, eTIV, or age in FEP subjects (hierarchy level 1).

| **Region** | **Regressor** | **β-coefficient** | **p-value** | **Significance** |
| --- | --- | --- | --- | --- |
| **Medial** | BPRS p | 0.578 | 0.229 |  |
|  | SANS | -0.693 | 0.161 |  |
|  | Phonemic fluency | -0.636 | 0.061 |  |

*BPRS Brief Psychiatric Rating Scale, FEP first-episode psychosis, p positive, SANS Scale for the Assessment of Negative Symptoms.*

Table S10.2.2: Association between medial nuclei volume and cognitive performance when accounting for sex in FEP subjects (hierarchy level 2).

| **Region** | **Regressor** | **β-coefficient** | **p-value** | **Significance** |
| --- | --- | --- | --- | --- |
| **Medial** | BPRS p | 0.755 | 0.177 |  |
|  | SANS | -0.838 | 0.134 |  |
|  | Phonemic fluency | -0.725 | 0.059 |  |
|  | Sex | -0.289 | 0.406 |  |

*BPRS Brief Psychiatric Rating Scale, FEP first-episode psychosis, p positive, SANS Scale for the Assessment of Negative Symptoms.*

Table S10.2.3: Association between medial nuclei volume and cognitive performance when accounting for sex and eTIV in FEP subjects (hierarchy level 3).

| **Region** | **Regressor** | **β-coefficient** | **p-value** | **Significance** |
| --- | --- | --- | --- | --- |
| **Medial** | BPRS p | 0.939 | 0.195 |  |
|  | SANS | -1.045 | 0.165 |  |
|  | Phonemic fluency | -0.805 | 0.081 |  |
|  | Sex | -0.523 | 0.378 |  |
|  | eTIV | -0.276 | 0.592 |  |

*BPRS Brief Psychiatric Rating Scale, eTIV estimated total intracranial volume, FEP first-episode psychosis, p positive, SANS Scale for the Assessment of Negative Symptoms.*

Table S10.2.4: Association between medial nuclei volume and cognitive performance measured with phonemic fluency when accounting for sex, eTIV, and age in FEP subjects (hierarchy level 4).

| **Region** | **Regressor** | **β-coefficient** | **p-value** | **Significance** |
| --- | --- | --- | --- | --- |
| **Medial** | BPRS p | 0.933 | 0.243 |  |
|  | SANS | -1.009 | 0.222 |  |
|  | Phonemic fluency | -0.760 | 0.136 |  |
|  | Sex | -0.233 | 0.756 |  |
|  | eTIV | -0.111 | 0.854 |  |
|  | Age | -0.316 | 0.515 |  |

*BPRS Brief Psychiatric Rating Scale, eTIV estimated total intracranial volume, FEP first-episode psychosis, p positive, SANS Scale for the Assessment of Negative Symptoms.*

### S10.3: Association between medial nuclei volume and phonemic fluency in SCZ

Table S10.3.1: Association between medial nuclei volume and cognitive performance measured with phonemic fluency when not accounting for sex, eTIV, or age in SCZ subjects (hierarchy level 1).

| **Region** | **Regressor** | **β-coefficient** | **p-value** | **Significance** |
| --- | --- | --- | --- | --- |
| **Medial** | PANSS p | -0.114 | 0.359 |  |
|  | PANSS n | 0.150 | 0.166 |  |
|  | Phonemic fluency | 0.025 | 0.843 |  |

*eTIV estimated total intracranial volume, n negative, PANSS* *Positive and Negative Syndrome Scale, p positive, SCZ schizophrenia.*

Table S10.3.2: Association between medial nuclei volume and cognitive performance measured as phonemic fluency when accounting for sex in SCZ subjects (hierarchy level 2).

| **Region** | **Regressor** | **β-coefficient** | **p-value** | **Significance** |
| --- | --- | --- | --- | --- |
| **Medial** | PANSS p | -0.115 | 0.362 |  |
|  | PANSS n | 0.149 | 0.177 |  |
|  | Phonemic fluency | 0.025 | 0.844 |  |
|  | Sex | -0.006 | 0.958 |  |

*eTIV estimated total intracranial volume, n negative, PANSS* *Positive and Negative Syndrome Scale, p positive, SCZ schizophrenia.*

Table S10.3.3: Association between medial nuclei volume and cognitive performance measured as phonemic fluency when accounting for sex and eTIV in SCZ subjects (hierarchy level 3).

| **Region** | **Regressor** | **β-coefficient** | **p-value** | **Significance** |
| --- | --- | --- | --- | --- |
| **Medial** | PANSS p | -0.176 | 0.145 |  |
|  | PANSS n | 0.156 | 0.135 |  |
|  | Phonemic fluency | -0.033 | 0.781 |  |
|  | Sex | 0.092 | 0.395 |  |
|  | eTIV | 0.358 | 0.001 | *** |

*eTIV estimated total intracranial volume, n negative, PANSS* *Positive and Negative Syndrome Scale, p positive, SCZ schizophrenia.*

Table S10.3.4: Association between medial nuclei volume and cognitive performance measured with phonemic fluency when accounting for sex, eTIV, and age in SCZ subjects (hierarchy level 4).

| **Subregion** | **Regressor** | **β-coefficient** | **p-value** | **Significance** |
| --- | --- | --- | --- | --- |
| **Medial** | PANSS p | -0.096 | 0.372 |  |
|  | PANSS n | 0.073 | 0.432 |  |
|  | Phonemic fluency | -0.091 | 0.389 |  |
|  | Sex | 0.129 | 0.177 |  |
|  | eTIV | 0.400 | <0.001 | *** |
|  | Age | -0.472 | <0.001 | *** |

*eTIV estimated total intracranial volume, n negative, PANSS* *Positive and Negative Syndrome Scale, p positive, SCZ schizophrenia.*

### S10.4: Association between medial nuclei volume and TMT-A in SCZ

Table S10.4.1: Association between medial nuclei volume and cognitive performance measured with TMT-A when not accounting for sex, eTIV, or age in SCZ subjects (hierarchy level 1).

| **Region** | **Regressor** | **β-coefficient** | **p-value** | **Significance** |
| --- | --- | --- | --- | --- |
| **Medial** | PANSS p | 0.018 | 0.872 |  |
|  | PANSS n | 0.145 | 0.226 |  |
|  | TMT-A | -0.425 | <0.001 | *** |

*eTIV estimated total intracranial volume, n negative, PANSS* *Positive and Negative Syndrome Scale, p positive, TMT-A Trail Making Test Part A, SCZ schizophrenia.*

Table S10.4.2: Association between medial nuclei volume and cognitive performance measured with TMT-A when accounting for sex in SCZ subjects (hierarchy level 2).

| **Region** | **Regressor** | **β-coefficient** | **p-value** | **Significance** |
| --- | --- | --- | --- | --- |
| **Medial** | PANSS p | 0.018 | 0.873 |  |
|  | PANSS n | 0.145 | 0.223 |  |
|  | TMT-A | -0.425 | <0.001 | *** |
|  | Sex | 0.001 | 0.993 |  |

*eTIV estimated total intracranial volume, n negative, PANSS* *Positive and Negative Syndrome Scale, p positive, TMT-A Trail Making Test Part A, SCZ schizophrenia.*

Table S10.4.3: Association between medial nuclei volume and cognitive performance measured with TMT-A when accounting for sex and eTIV in SCZ subjects (hierarchy level 3).

| **Region** | **Regressor** | **β-coefficient** | **p-value** | **Significance** |
| --- | --- | --- | --- | --- |
| **Medial** | PANSS p | -0.011 | 0.915 |  |
|  | PANSS n | 0.142 | 0.219 |  |
|  | TMT-A | -0.386 | 0.001 | *** |
|  | Sex | 0.115 | 0.319 |  |
|  | eTIV | 0.323 | 0.006 | ** |

*eTIV estimated total intracranial volume, n negative, PANSS* *Positive and Negative Syndrome Scale, p positive, TMT-A Trail Making Test Part A, SCZ schizophrenia.*

Table S10.4.4: Association between medial nuclei volume and cognitive performance measured with TMT-A when accounting for sex, eTIV, and age in SCZ subjects (model 4).

| **Region** | **Regressor** | **β-coefficient** | **p-value** | **Significance** |
| --- | --- | --- | --- | --- |
| **Medial** | PANSS p | -0.012 | 0.890 |  |
|  | PANSS n | -0.025 | 0.802 |  |
|  | TMT-A | -0.160 | 0.127 |  |
|  | Sex | 0.114 | 0.234 |  |
|  | eTIV | 0.362 | <0.001 | *** |
|  | Age | -0.543 | <0.001 | *** |

*eTIV estimated total intracranial volume, n negative, PANSS* *Positive and Negative Syndrome Scale, p positive, TMT-A Trail Making Test Part A, SCZ schizophrenia.*

### S10.5: Association between medial nuclei volume and SCT in SCZ

Table S10.5.1: Association between medial nuclei volume and cognitive performance measured with SCT when not accounting for sex, eTIV, or age in SCZ subjects (hierarchy level 1).

| **Region** | **Regressor** | **β-coefficient** | **p-value** | **Significance** |
| --- | --- | --- | --- | --- |
| **Medial** | PANSS p | 0.031 | 0.769 |  |
|  | PANSS n | 0.097 | 0.366 |  |
|  | SCT | 0.447 | <0.001 | *** |

*eTIV estimated total intracranial volume, n negative, PANSS* *Positive and Negative Syndrome Scale, p positive, SCT* *Symbol Coding Task of the Brief Assessment of Cognition in Schizophrenia, SCZ schizophrenia.*

Table S10.5.2: Association between nuclei volume and cognitive performance measured with SCT when accounting for sex in SCZ subjects (hierarchy level 2).

| **Region** | **Regressor** | **β-coefficient** | **p-value** | **Significance** |
| --- | --- | --- | --- | --- |
| **Medial** | PANSS p | 0.027 | 0.797 |  |
|  | PANSS n | 0.095 | 0.383 |  |
|  | SCT | 0.446 | <0.001 | *** |
|  | Sex | -0.026 | 0.809 |  |

*eTIV estimated total intracranial volume, n negative, PANSS* *Positive and Negative Syndrome Scale, p positive, SCT* *Symbol Coding Task of the Brief Assessment of Cognition in Schizophrenia, SCZ schizophrenia.*

Table S10.5.3: Association between medial nuclei volume and cognitive performance measured with SCT when accounting for sex and eTIV in SCZ subjects (hierarchy level 3).

| **Region** | **Regressor** | **β-coefficient** | **p-value** | **Significance** |
| --- | --- | --- | --- | --- |
| **Medial** | PANSS p | -0.009 | 0.925 |  |
|  | PANSS n | 0.108 | 0.288 |  |
|  | SCT | 0.443 | <0.001 | *** |
|  | Sex | 0.111 | 0.299 |  |
|  | eTIV | 0.365 | <0.001 | *** |

*eTIV estimated total intracranial volume, n negative, PANSS* *Positive and Negative Syndrome Scale, p positive, SCT* *Symbol Coding Task of the Brief Assessment of Cognition in Schizophrenia, SCZ schizophrenia.*

Table S10.5.4: Association between medial nuclei volume and cognitive performance measured with SCT when accounting for sex, eTIV, and age in SCZ subjects (hierarchy level 4).

| **Region** | **Regressor** | **β-coefficient** | **p-value** | **Significance** |
| --- | --- | --- | --- | --- |
| **Medial** | PANSS p | -0.026 | 0.770 |  |
|  | PANSS n | -0.019 | 0.844 |  |
|  | SCT | 0.156 | 0.158 |  |
|  | Sex | 0.118 | 0.216 |  |
|  | eTIV | 0.380 | <0.001 | *** |
|  | Age | -0.482 | <0.001 | *** |

*eTIV estimated total intracranial volume, n negative, PANSS* *Positive and Negative Syndrome Scale, p positive, SCT* *Symbol Coding Task of the Brief Assessment of Cognition in Schizophrenia, SCZ schizophrenia.*

### S10.6: Association between lateral nuclei volume and phonemic fluency in SCZ

Table S10.6.1: Association between lateral nuclei volume and cognitive performance measured with phonemic fluency when not accounting for sex, eTIV, or age in SCZ subjects (hierarchy level 1).

| **Region** | **Regressor** | **β-coefficient** | **p-value** | **Significance** |
| --- | --- | --- | --- | --- |
| **Lateral** | PANSS p | -0.046 | 0.709 |  |
|  | PANSS n | 0.018 | 0.868 |  |
|  | Phonemic fluency | 0.193 | 0.123 |  |

*eTIV estimated total intracranial volume, n negative, PANSS* *Positive and Negative Syndrome Scale, p positive, SCZ schizophrenia.*

Table S10.6.2: Association between lateral nuclei volume and cognitive performance measured with phonemic fluency when accounting for sex in SCZ subjects (hierarchy level 2).

| **Region** | **Regressor** | **β-coefficient** | **p-value** | **Significance** |
| --- | --- | --- | --- | --- |
| **Lateral** | PANSS p | -0.060 | 0.627 |  |
|  | PANSS n | 0.002 | 0.985 |  |
|  | Phonemic fluency | 0.192 | 0.125 |  |
|  | Sex | -0.103 | 0.346 |  |

*eTIV estimated total intracranial volume, n negative, PANSS* *Positive and Negative Syndrome Scale, p positive, SCZ schizophrenia.*

Table S10.6.3: Association between lateral nuclei volume and cognitive performance measured with phonemic fluency when accounting for sex and eTIV in SCZ subjects (hierarchy level 3).

| **Region** | **Regressor** | **β-coefficient** | **p-value** | **Significance** |
| --- | --- | --- | --- | --- |
| **Lateral** | PANSS p | -0.108 | 0.375 |  |
|  | PANSS n | 0.007 | 0.944 |  |
|  | Phonemic fluency | 0.146 | 0.230 |  |
|  | Sex | -0.026 | 0.809 |  |
|  | eTIV | 0.280 | 0.012 | * |

*eTIV estimated total intracranial volume, n negative, PANSS* *Positive and Negative Syndrome Scale, p positive, TMT-A Trail Making Test Part A, SCZ schizophrenia.*

Table S10.6.4: Association between lateral nuclei volume and cognitive performance measured with phonemic fluency when accounting for sex, eTIV, and age in SCZ subjects (hierarchy level 4).

| **Region** | **Regressor** | **β-coefficient** | **p-value** | **Significance** |
| --- | --- | --- | --- | --- |
|  | PANSS p | -0.061 | 0.610 |  |
|  | PANSS n | -0.042 | 0.686 |  |
|  | Phonemic fluency | 0.112 | 0.343 |  |
|  | Sex | -0.004 | 0.967 |  |
|  | eTIV | 0.305 | 0.005 | ** |
|  | Age | -0.279 | 0.009 | ** |

*eTIV estimated total intracranial volume, n negative, PANSS* *Positive and Negative Syndrome Scale, p positive, SCZ schizophrenia.*

### S10.7: Association between lateral nuclei volume and TMT-A in SCZ

Table S10.7.1: Association between lateral nuclei volume and cognitive performance measured with TMT-A when not accounting for sex, eTIV, or age in SCZ subjects (hierarchy level 1).

| **Region** | **Regressor** | **β-coefficient** | **p-value** | **Significance** |
| --- | --- | --- | --- | --- |
| **Lateral** | PANSS p | -0.085 | 0.465 |  |
|  | PANSS n | 0.021 | 0.869 |  |
|  | TMT-A | -0.224 | 0.077 |  |

*eTIV estimated total intracranial volume, n negative, PANSS* *Positive and Negative Syndrome Scale, p positive, TMT-A Trail Making Test Part A, SCZ schizophrenia.*

Table S10.7.2: Association between lateral nuclei volume and cognitive performance measured with TMT-A when accounting for sex in SCZ subjects (hierarchy level 2).

| **Region** | **Regressor** | **β-coefficient** | **p-value** | **Significance** |
| --- | --- | --- | --- | --- |
| **Lateral** | PANSS p | -0.103 | 0.378 |  |
|  | PANSS n | 0.002 | 0.985 |  |
|  | TMT-A | -0.203 | 0.113 |  |
|  | Sex | -0.139 | 0.242 |  |

*eTIV estimated total intracranial volume, n negative, PANSS* *Positive and Negative Syndrome Scale, p positive, TMT-A Trail Making Test Part A, SCZ schizophrenia.*

Table S10.7.3: Association between lateral nuclei volume and cognitive performance measured with TMT-A when accounting for sex and eTIV in SCZ subjects (hierarchy level 3).

| **Region** | **Regressor** | **β-coefficient** | **p-value** | **Significance** |
| --- | --- | --- | --- | --- |
| **Lateral** | PANSS p | -0.118 | 0.313 |  |
|  | PANSS n | 0.001 | 0.994 |  |
|  | TMT-A | -0.182 | 0.153 |  |
|  | Sex | -0.080 | 0.523 |  |
|  | eTIV | 0.166 | 0.190 |  |

*eTIV estimated total intracranial volume, n negative, PANSS* *Positive and Negative Syndrome Scale, p positive, TMT-A Trail Making Test Part A, SCZ schizophrenia.*

Table S10.7.4: Association between lateral nuclei volume and cognitive performance measured with TMT-A when accounting for sex, eTIV, and age in SCZ subjects (hierarchy level 4).

| **Region** | **Regressor** | **β-coefficient** | **p-value** | **Significance** |
| --- | --- | --- | --- | --- |
| **Lateral** | PANSS p | -0.119 | 0.288 |  |
|  | PANSS n | -0.104 | 0.408 |  |
|  | TMT-A | -0.041 | 0.754 |  |
|  | Sex | -0.081 | 0.499 |  |
|  | eTIV | 0.190 | 0.116 |  |
|  | Age | -0.340 | 0.006 | ** |

*eTIV estimated total intracranial volume, n negative, PANSS* *Positive and Negative Syndrome Scale, p positive, TMT-A Trail Making Test Part A, SCZ schizophrenia.*

### S10.8: Association between lateral nuclei volume and SCT in SCZ

Table S10.8.1: Association between lateral nuclei volume and cognitive performance measured with SCT when not accounting for sex, eTIV, or age in SCZ subjects (hierarchy level 1).

| **Region** | **Regressor** | **β-coefficient** | **p-value** | **Significance** |
| --- | --- | --- | --- | --- |
| **Lateral** | PANSS p | -0.079 | 0.482 |  |
|  | PANSS n | -0.020 | 0.861 |  |
|  | SCT | 0.203 | 0.083 |  |

*eTIV estimated total intracranial volume, n negative, PANSS* *Positive and Negative Syndrome Scale, p positive, SCT* *Symbol Coding Task of the Brief Assessment of Cognition in Schizophrenia, SCZ schizophrenia.*

Table S10.8.2: Association between lateral nuclei volume and cognitive performance measured with SCT when accounting for sex in SCZ subjects (hierarchy level 2).

| **Region** | **Regressor** | **β-coefficient** | **p-value** | **Significance** |
| --- | --- | --- | --- | --- |
| **Lateral** | PANSS p | -0.101 | 0.369 |  |
|  | PANSS n | -0.035 | 0.761 |  |
|  | SCT | 0.195 | 0.094 |  |
|  | Sex | -0.164 | 0.149 |  |

*eTIV estimated total intracranial volume, n negative, PANSS* *Positive and Negative Syndrome Scale, p positive, SCT* *Symbol Coding Task of the Brief Assessment of Cognition in Schizophrenia, SCZ schizophrenia.*

Table S10.8.3: Association between lateral nuclei volume and cognitive performance measured with SCT when accounting for sex and eTIV in SCZ subjects (hierarchy level 3).

| **Region** | **Regressor** | **β-coefficient** | **p-value** | **Significance** |
| --- | --- | --- | --- | --- |
| **Lateral** | PANSS p | -0.120 | 0.286 |  |
|  | PANSS n | -0.028 | 0.804 |  |
|  | SCT | 0.193 | 0.094 |  |
|  | Sex | -0.094 | 0.436 |  |
|  | eTIV | 0.187 | 0.123 |  |

*eTIV estimated total intracranial volume, n negative, PANSS* *Positive and Negative Syndrome Scale, p positive, SCT* *Symbol Coding Task of the Brief Assessment of Cognition in Schizophrenia, SCZ schizophrenia.*

Table S10.8.4: Association between lateral nuclei volume and cognitive performance measured with SCT when accounting for sex, eTIV, and age in SCZ subjects (hierarchy level 4).

| **Region** | **Regressor** | **β-coefficient** | **p-value** | **Significance** |
| --- | --- | --- | --- | --- |
| **Lateral** | PANSS p | -0.131 | 0.232 |  |
|  | PANSS n | -0.111 | 0.342 |  |
|  | SCT | 0.007 | 0.960 |  |
|  | Sex | -0.090 | 0.444 |  |
|  | eTIV | 0.197 | 0.095 |  |
|  | Age | -0.314 | 0.020 | * |

*eTIV estimated total intracranial volume, n negative, PANSS Positive and Negative Syndrome Scale, p positive, SCT Symbol Coding Task of the Brief Assessment of Cognition in Schizophrenia.*

## Table S11: Control analysis: hierarchical multiple linear regression analysis of cognitive impairments and thalamic nuclei volumes in healthy controls

Table S11.1: Association between medial and lateral nuclei volume and cognitive performance measured with animal fluency when not accounting for sex, eTIV, or age in HC subjects (hierarchy level 1).

| **Region** | **Regressor** | **β-coefficient** | **p-value** | **Significance** |
| --- | --- | --- | --- | --- |
| **Medial** | Animal fluency | 0.038 | 0.758 |  |
| **Lateral** | Animal fluency | -0.148 | 0.231 |  |

*HC healthy controls.*

Table S11.2: Association between medial and lateral nuclei volume and cognitive performance measured with phonemic fluency when not accounting for sex, eTIV, or age in HC subjects (hierarchy level 1).

| **Region** | **Regressor** | **β-coefficient** | **p-value** | **Significance** |
| --- | --- | --- | --- | --- |
| **Medial** | Phonemic fluency | 0.002 | 0.990 |  |
| **Lateral** | Phonemic fluency | 0.136 | 0.272 |  |

*HC healthy controls.*

Table S11.3: Association between medial and lateral nuclei volume and cognitive performance measured with TMT-A when not accounting for sex, eTIV, or age in HC subjects (hierarchy level 1).

| **Region** | **Regressor** | **β-coefficient** | **p-value** | **Significance** |
| --- | --- | --- | --- | --- |
| **Medial** | TMT-A | -0.008 | 0.951 |  |
| **Lateral** | TMT-A | -0.057 | 0.648 |  |

*HC healthy controls, TMT-A Trail Making Test Part A, SCZ schizophrenia.*

Table S11.4: Association between medial and lateral nuclei volume and cognitive performance measured with SCT when not accounting for sex, eTIV, or age in HC subjects (hierarchy level 1).

| **Region** | **Regressor** | **β-coefficient** | **p-value** | **Significance** |
| --- | --- | --- | --- | --- |
| **Medial** | SCT | 0.187 | 0.130 |  |
| **Lateral** | SCT | 0.115 | 0.356 |  |

*HC healthy controls, SCT* *Symbol Coding Task of the Brief Assessment of Cognition in Schizophrenia.*

# Supplementary figures


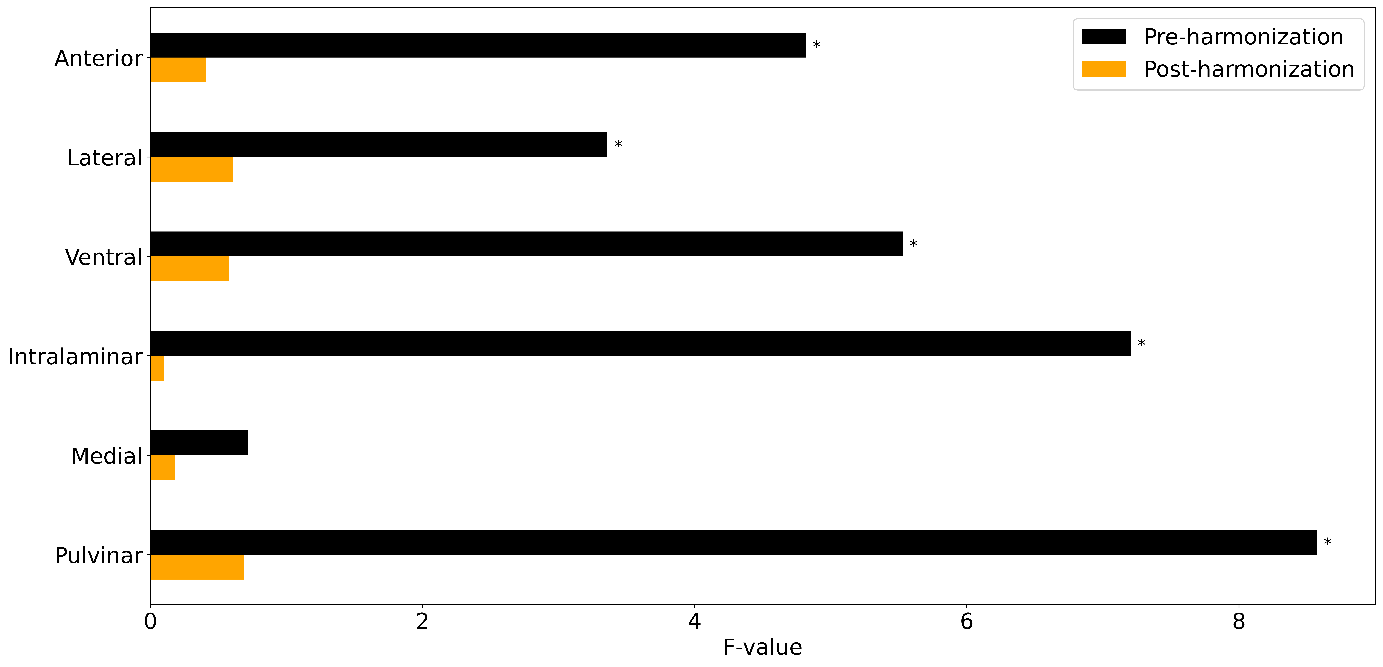


**Figure S1: Harmonization effect.** Differences in regional thalamus nuclei volumes between scanners were assessed prior and after harmonization using NeuroCombat. Healthy controls across sites were compared in an Analysis of Covariance (ANCOVA), with regional thalamus nuclei volume as dependent variable and age, sex, and eTIV (uncorrected or corrected for scanner effects, respectively) as covariates. Significant (p_unc_<0.05) differences across sites were marked with an asterisk (*).

References

1. Weeland CJ, Kasprzak S, Joode NT de, et al. The thalamus and its subnuclei-a gateway to obsessive-compulsive disorder. *Transl Psychiatry*. 2022;12(1):70. doi:10.1038/s41398-022-01823-2.

2. Sämann PG, Iglesias JE, Gutman B, et al. FreeSurfer-based segmentation of hippocampal subfields: A review of methods and applications, with a novel quality control procedure for ENIGMA studies and other collaborative efforts. *Hum Brain Mapp*. 2022;43(1):207-233. doi:10.1002/hbm.25326.

3. Vallat R. Pingouin: statistics in Python. *JOSS*. 2018;3(31):1026. doi:10.21105/joss.01026.

4. Virtanen P, Gommers R, Oliphant TE, et al. SciPy 1.0: fundamental algorithms for scientific computing in Python. *Nat Methods*. 2020;17(3):261-272. doi:10.1038/s41592-019-0686-2.

5. Hayes AF. *Introduction to mediation, moderation, and conditional process analysis: A regression-based approach.* Third edition. New York NY: The Guilford Press; 2022. Methodology in the social sciences.

6. Richter S, Winzeck S, Correia MM, et al. Validation of cross-sectional and longitudinal ComBat harmonization methods for magnetic resonance imaging data on a travelling subject cohort. *Neuroimage Rep*. 2022;2(4). doi:10.1016/j.ynirp.2022.100136.

7. Bayer JMM, Thompson PM, Ching CRK, et al. Site effects how-to and when: An overview of retrospective techniques to accommodate site effects in multi-site neuroimaging analyses. *Front Neurol*. 2022;13:923988. doi:10.3389/fneur.2022.923988.

8. Opasso PR, Barreto SDS, Ortiz KZ. Phonemic verbal fluency task in adults with high-level literacy. *Einstein (Sao Paulo)*. 2016;14(3). doi:10.1590/S1679-45082016AO3629.

9. Ruff RM, Light RH, Parker SB, Levin HS. The psychological construct of word fluency. *Brain Lang*. 1997;57(3):394-405. doi:10.1006/brln.1997.1755.

10. Shao Z, Janse E, Visser K, Meyer AS. What do verbal fluency tasks measure? Predictors of verbal fluency performance in older adults. *Front Psychol*. 2014;5:772. doi:10.3389/fpsyg.2014.00772.

11. Spets DS, Slotnick SD. Thalamic Functional Connectivity during Spatial Long-Term Memory and the Role of Sex. *Brain Sci*. 2020;10(12). doi:10.3390/brainsci10120898.

12. Parnaudeau S, Bolkan SS, Kellendonk C. The Mediodorsal Thalamus: An Essential Partner of the Prefrontal Cortex for Cognition. *Biol Psychiatry*. 2018;83(8):648-656. doi:10.1016/j.biopsych.2017.11.008.

13. Tombaugh TN. Trail Making Test A and B: normative data stratified by age and education. *Arch Clin Neuropsychol*. 2004;19(2):203-214. doi:10.1016/S0887-6177(03)00039-8.

14. Varjacic A, Mantini D, Demeyere N, Gillebert CR. Neural signatures of Trail Making Test performance: Evidence from lesion-mapping and neuroimaging studies. *Neuropsychologia*. 2018;115:78-87. doi:10.1016/j.neuropsychologia.2018.03.031.

15. Hwang K, Bruss J, Tranel D, Boes AD. Network Localization of Executive Function Deficits in Patients with Focal Thalamic Lesions. *J Cogn Neurosci*. 2020;32(12):2303-2319. doi:10.1162/jocn_a_01628.

16. Keefe RSE, Goldberg TE, Harvey PD, Gold JM, Poe MP, Coughenour L. The Brief Assessment of Cognition in Schizophrenia: reliability, sensitivity, and comparison with a standard neurocognitive battery. *Schizophr Res*. 2004;68(2-3):283-297. doi:10.1016/j.schres.2003.09.011.

17. Bisecco A, Stamenova S, Caiazzo G, et al. Attention and processing speed performance in multiple sclerosis is mostly related to thalamic volume. *Brain Imaging Behav*. 2018;12(1):20-28. doi:10.1007/s11682-016-9667-6.

18. Bergsland N, Zivadinov R, Dwyer MG, Weinstock-Guttman B, Benedict RH. Localized atrophy of the thalamus and slowed cognitive processing speed in MS patients. *Mult Scler*. 2016;22(10):1327-1336. doi:10.1177/1352458515616204.

19. Takahashi T, Tsugawa S, Nakajima S, et al. Thalamic and striato-pallidal volumes in schizophrenia patients and individuals at risk for psychosis: A multi-atlas segmentation study. *Schizophr Res*. 2022;243:268-275. doi:10.1016/j.schres.2020.04.016.
